# Establishment and functional analysis of an inducible *Apoc2-*knockout mouse model to investigate the role of *Apoc2* in normal hematopoiesis

**DOI:** 10.1038/s41684-026-01748-z

**Published:** 2026-07-10

**Authors:** Xiaochen Zhang, Ritu Chandwani, Mateusz Pospiech, Yiting Meng, Kanaka Dhuri, Atham Ali, Bhakti Thakker, Shephali Milind Kadam, Wenda Zhu, Yiyu Xiao, Denis Sviridov, Anna Wolska, Alan T. Remaley, Houda Alachkar

**Affiliations:** 1 Department of Clinical Pharmacy, Alfred E. Mann School of Pharmacy and Pharmaceutical Sciences, University of Southern California, Los Angeles, CA USA; 2 Lipoprotein Metabolism Laboratory, Translational Vascular Medicine Branch, National Heart, Lung, and Blood Institute, National Institutes of Health, Bethesda, MD USA; 3 USC Norris Comprehensive Cancer Center, University of Southern California, Los Angeles, CA USA

**Keywords:** Animal breeding, Haematopoiesis

## Abstract

Apolipoprotein C2 (APOC2) has been identified as a potential therapeutic target for acute myeloid leukemia, but its impact on normal hematopoiesis remains unclear. Here we generated a global inducible *Apoc2* knockout (KO) mouse model by breeding *R26-CreERT2* mice with mice homozygous for *Apoc2* *lox* sites (*Apoc2*^*fl/fl*^) over three generations. Global *Apoc2* KO was induced in 4–8-week-old mice by 5 consecutive days of intraperitoneal administration of tamoxifen. Successful *Apoc2* KO was confirmed by measuring *Apoc2* mRNA levels, showing 80–99% reduction in the liver, bone marrow, spleen and blood tissues compared with wild type (WT). Despite significantly higher serum triglyceride levels in the *Apoc2-*KO group, no apparent abnormal phenotypes were observed. In vitro expansion of the hematopoietic stem and progenitor cells and splenocytes from *Apoc2*-KO were comparable to that of WT mice. In addition, *Apoc2* deletion did not significantly affect the colony-forming ability or the competitive repopulation ability of hematopoietic stem and progenitor cells. Hematological analysis revealed no significant differences between *Apoc2-*KO and WT mice. RNA-sequencing analysis of bone marrow samples from the *Apoc2-*KO group identified four significantly downregulated genes and five upregulated genes, including *Gm16867*, which is orthologous to the human *SLC25A37* gene involved in mitochondrial iron homeostasis. Gene set enrichment analysis revealed the enrichment of heme metabolism (false discovery rate 0.077). Our study demonstrates that deletion of *Apoc2* exhibits limited impact on normal hematopoiesis under normal conditions. Future characterization of this Cre-inducible *Apoc2*-KO mice under stress conditions or during aging is needed to fully demonstrate the biological impact of *Apoc2* deletion.

## Abstract (web summary)

This study shows that inducible global deletion of *Apoc2* in mice causes hypertriglyceridemia but has minimal impact on normal hematopoiesis, supporting APOC2 as a potential therapeutic target with limited baseline hematopoietic risk.

## Main

Acute myeloid leukemia (AML) is a hematologic malignancy arising in the bone marrow (BM) and is characterized by uncontrolled proliferation of immature myeloid cells^1,2^. Our previous research revealed that apolipoprotein C2 (*APOC2*) messenger RNA levels were significantly upregulated in AML, probably to satisfy the increased energy demands associated with the proliferation of myeloid leukemia cells^3^. APOC2 cooperates with CD36, which functions as a fatty acid translocase and a scavenger receptor, to promote leukemia growth by activating the ERK signaling pathway. Knocking down APOC2 in leukemia cells decreased cell proliferation and induced apoptosis, suggesting APOC2 as a potential therapeutic target for AML treatment^3^. However, the effects of targeting APOC2 on healthy hematopoietic cells remain unclear.

APOC2, a subtype of apolipoprotein C, is an important modulator of lipoprotein metabolism and is found on chylomicrons, very low-density lipoproteins (VLDL) and high-density lipoproteins (HDL)^4,5^. The human *APOC2* gene is located on chromosome 19q13.32 within the *APOE*–*APOC1*–*APOC4*–*APOC2* gene cluster (48 kb) and is predominantly expressed in the liver^4,5^. APOC2 has a key role in lipid delivery and metabolism by activating lipoprotein lipase (LPL), the main plasma enzyme that hydrolyzes triglycerides (TGs)^6,7^. The free fatty acids generated during lipolysis are taken up by cells^4^ and undergo oxidation and serve as an important energy source for cells^3,6^. The upregulation of *APOC2* in several malignancies has been found to be associated with poor overall survival^8^. Mutations in *APOC2* gene are common in patients with familial chylomicronemia syndrome, which is characterized by hypertriglyceridemia and recurrent acute pancreatitis^4,9^.

Several animal models have been developed to study APOC2 deficiency. *Apoc2-*knockout (KO) rodent models and *Apoc2-*mutant mouse models have been generated using various techniques, including CRISPR–Cas9^10^ and zinc finger nucleases^11^ to edit fertilized oocytes. However, *Apoc2*-deficient rodents displayed a neonatal lethal phenotype^10,11^, limiting the use of these animal models. Thus, the inducible Cre/*lox* system offers an effective approach for deleting the *Apoc2* gene in mice. This strategy allows one to study the effects of *Apoc2* deletion at specific developmental stages or in particular tissues, thereby avoiding complications, such as embryonic or neonatal lethality or systemic effects that could obscure the role of the gene in specific biological processes^12^.

In this study, to assess the effects of *Apoc2* deletion on normal hematopoiesis, we established and characterized an inducible and global *Apoc2*-KO mouse model based on the use of the Cre/*lox* system.

## Results

### Generation of the Cre-inducible *Apoc2-KO* mouse model

**Fig. 1:**
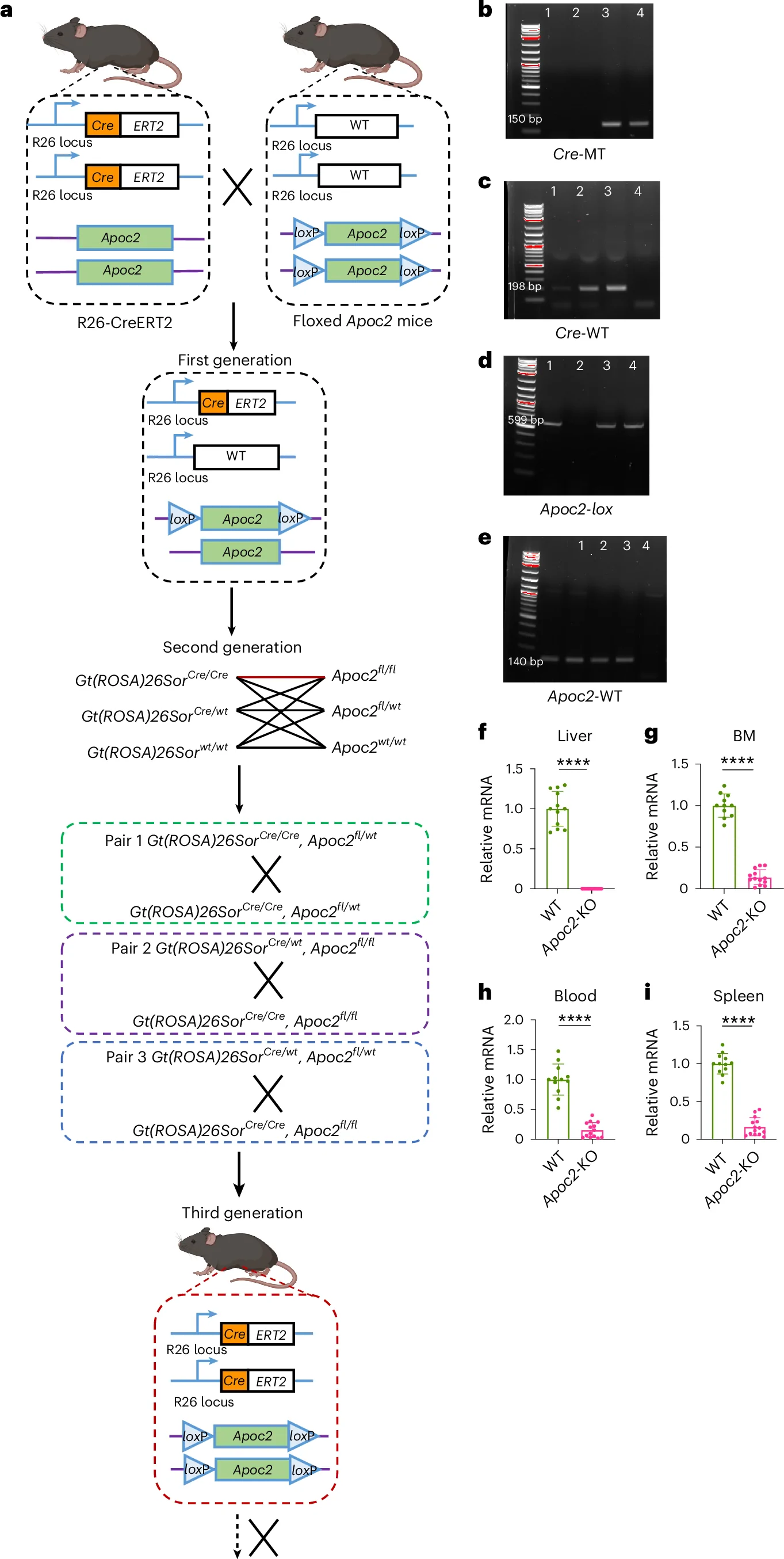
Generation of Cre-inducible *Apoc2*-KO mouse model. **a**, *Gt(ROSA)26Sor*^*Cre/Cre*^*Apoc2*^*fl/fl*^ mice breeding scheme. *Gt(ROSA)26Sor*^*Cre/Cre*^*Apoc2*^*wt/wt*^ mice were crossed with *Gt(ROSA)26Sor*^*wt/wt*^*Apoc2*^*fl/fl*^ mice to get the first generation of *Gt(ROSA)26Sor*^*Cre/wt*^*Apoc2*^*fl/wt*^. Nine genotypes were obtained in the second generation, including two male *Gt(ROSA)26Sor*^*Cre/Cre*^*Apoc2*^*fl/fl*^ mice. To increase the odds and shorten the time of breeding homozygous mice in the third generation, three new breeding pairs were designed. *Gt(ROSA)26Sor*^*Cre/wt*^*Apoc2*^*fl/fl*^ female mouse was crossed with *Gt(ROSA)26Sor*^*Cre/Cre*^*Apoc2*^*fl/fl*^ male mouse. *Gt(ROSA)26Sor*^*Cre/Cre*^*Apoc2*^*fl/wt*^ female mouse was crossed with *Gt(ROSA)26Sor*^*Cre/Cre*^*Apoc2*^*fl/wt*^ male mouse, and *Gt(ROSA)26Sor*^*Cre/wt*^*Apoc2*^*fl/wt*^ female mouse was crossed with *Gt(ROSA)26Sor*^*Cre/Cre*^*Apoc2*^*fl/fl*^ male mouse. Female and male *Gt(ROSA)26Sor*^*Cre/Cre*^*Apoc2*^*fl/fl*^ mice in the third generation were selected by genotyping and designated as breeding pairs to obtain homozygous mice in the following generations. **b**–**e**, Agarose gel electrophoresis showing four distinct genotypes across representative samples 1, *Gt(ROSA)26Sor*^*wt/wt*^*Apoc2*^*fl/wt*^; 2, *Gt(ROSA)26Sor*^*wt/wt*^*Apoc2*^*wt/wt*^; 3, *Gt(ROSA)26Sor*^*Cre/wt*^*Apoc2*^*fl/wt*^; 4, *Gt(ROSA)26Sor*^*Cre/Cre*^*Apoc2*^*fl/fl*^: PCR amplification using *Cre*-MT primers yielded a 150-bp product (**b**); PCR amplification using *Cre*-WT primers yielded a 198-bp product (**c**); PCR amplification using *Apoc2-lox* primers yielded a 599-bp product (**d**); PCR amplification using *Apoc2-*WT primers yielded a 140-bp product (**e**). **f**–**i**, qPCR quantification of *Apoc2* gene KO efficiency in *Apoc2*-KO mice compared with WT mice from experiments 1–7 (Supplementary Table 1). The WT group included three male CD45.2^+^ WT mice injected with TAM and nine *Gt(ROSA)26Sor*^*wt/wt*^*Apoc2*^*fl/fl*^ mice injected with TAM (*n* = 4 M + 5 F). The waiting period after injection ranged from 11 days to 5 months in the *Apoc2*-KO group and from 11 days to 4 months in the WT group. Tissues analyzed included liver tissues (*n* = 14 in *Apoc2*-KO group, *n* = 12 in WT group, *P* < 0.0001) (**f**), BM tissues (*n* = 13 in *Apoc2*-KO group, *n* = 11 in WT group, *P* < 0.0001) (**g**), blood (*n* = 14 in *Apoc2*-KO group, *n* = 12 in WT group, *P* < 0.0001) (**h**) and spleen tissues (n = 14 in *Apoc2*-KO group, *n* = 12 in WT group, *P* < 0.0001) (**i**). Shapiro–Wilk test was used to test the normality of data. Statistical analysis was done by unpaired two-tailed *t*-test (****P < 0.0001). Data are presented as mean ± s.d., and each dot represents a mouse. Mouse icon created in BioRender; Pospiech, M. https://biorender.com/hx8jbwn (2026). Source data

*Gt(ROSA)26Sor*^*Cre/Cre*^ mice were crossed with *Apoc2*^*fl/fl*^ mice to produce first-generation offspring, which were subsequently bred to generate second and third generations (Fig. 1a). Blood samples for DNA extraction were collected from the tail veins of mice across all generations. Polymerase chain reaction (PCR) genotyping used *Cre* and *Apoc2-*specific primers (Supplementary Fig. 1), revealing bands of 150 bp and 198 bp for the Cre-positive (mutant, *Cre*-MT) and Cre-negative (wild type, *Cre*-WT); and 599 bp and 140 bp for *Apoc2-lox* and *Apoc2-*WT, respectively (Fig. 1b–e).

First-generation mice were heterozygous for both *Cre* and *Apoc2 lox* sites (*Gt(ROSA)26Sor*^*Cre/wt*^*Apoc2*^*fl/wt*^). To enhance the yield of homozygous mice in subsequent generations, selected second-generation mice formed three breeding pairs. Following genotype confirmation, homozygous third-generation mice were bred to sustain homozygosity in future generations (Fig. 1a). All nine theoretical genotypes were observed (*Gt(ROSA)26Sor*^*wt/wt*^*Apoc2*^*wt/wt*^, *Gt(ROSA)26Sor*^*wt/wt*^*Apoc2*^*fl/wt*^, *Gt(ROSA)26Sor*^*wt/wt*^*Apoc2*^*fl/fl*^, *Gt(ROSA)26Sor*^*Cre/wt*^*Apoc2*^*wt/wt*^, *Gt(ROSA)26Sor*^*Cre*/*wt*^*Apoc2*^*fl/wt*^, *Gt(ROSA)26Sor*^*Cre/wt*^*Apoc2*^*fl/fl*^, *Gt(ROSA)26Sor*^*Cre*/*Cre*^*Apoc2*^*wt/wt*^, *Gt(ROSA)26Sor*^*Cre/Cre*^*Apoc2*^*fl/wt*^ and *Gt(ROSA)26Sor*^*Cre/Cre*^*Apoc2*^*fl/fl*^), with specific examples shown in Fig. 1b–e.

To confirm *Apoc2* deletion, relative *Apoc2* mRNA levels were measured in homozygous KO mice and compared with those in wild-type (WT) mice by quantitative real-time PCR in liver, spleen, BM and blood tissues. The shortest waiting period after the final tamoxifen (TAM) injection was 11 days, after which a 4–8-week-old male and female mice in the *Apoc2-*KO group were euthanized and levels of *Apoc2* expression in liver, BM, blood and spleen tissues were compared with that in WT mice. The average *Apoc2* mRNA levels across *Apoc2-*KO mice (experiments 1–7) showed a significant reduction compared with WT mice (*P* < 0.0001) in liver (99.97%; Fig. 1f), BM (86.32%; Fig. 1g), blood (84.60%; Fig. 1h) and spleen (83.41%; Fig. 1i). In WT mice (experiments 1–7), *Apoc2* mRNA levels in BM, blood and spleen tissues were significantly lower than those in liver tissues (*P* < 0.0001; Supplementary Fig. 2a), potentially explaining the lower KO efficiency observed in these tissues by quantitative PCR (qPCR). Analyses of the BloodSpot normal human hematopoiesis (DMAP) database shows that *APOC2* expression is comparable across the different blood cell types (Supplementary Fig. 2b).

### Body and tissue weights were not significantly different between WT and *Apoc2-*KO mice

Multiple control groups: *Gt(ROSA)26Sor*^*wt/wt*^*Apoc2*^*fl/fl*^ mice, *Gt(ROSA)26Sor*^*Cre/Cre*^*Apoc2*^*wt/wt*^ mice and C57BL/6J mice injected with TAM and *Gt(ROSA)26Sor*^*Cre/Cre*^*Apoc2*^*fl/fl*^ mice injected with vehicle were included to isolate the effects of *Apoc2* KO from those of TAM, Cre expression or genetic background. Except for the known side effect of TAM in male mice involving scrotal swelling affecting some males injected with TAM^13,14^ in both the *Apoc2-*KO group and the WT group, no obvious diseases or abnormal behaviors were observed in *Apoc2-*KO mice.

**Fig. 2:**
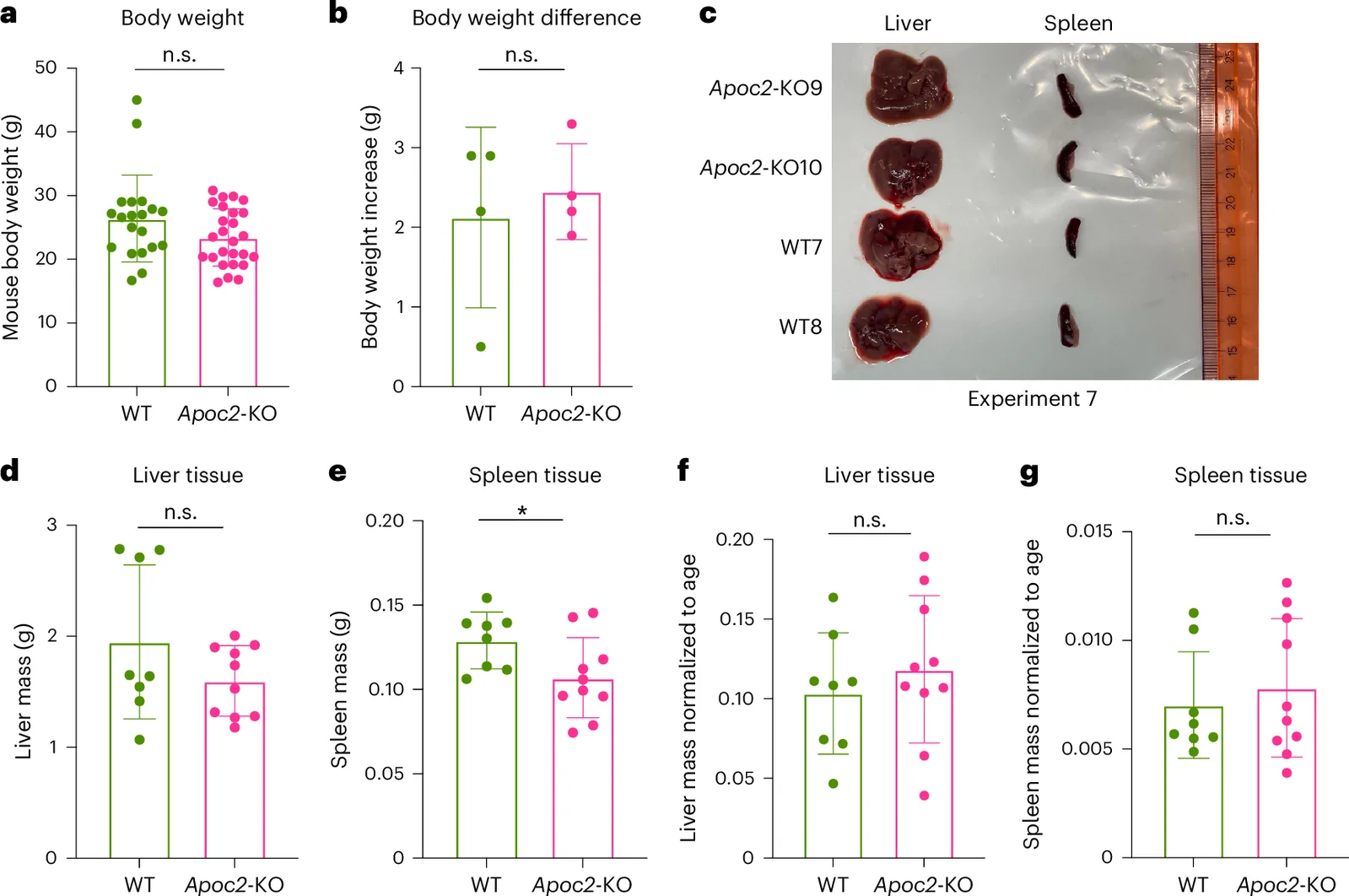
Comparison of body and organ weight and organ morphology in *Apoc2-*KO and WT mice. **a**, Body weight data for *Apoc2-*KO (*n* = 26) and WT mice (*n* = 22) were collected pre-euthanasia or 7+ days post-final injection (experiments 1, 5, 6 and 8 and live mice that were injected but not included in any experiment). Shapiro–Wilk test was used to test the normality of data, and Mann–Whitney test was used to compare the groups (*P* = 0.173). **b**, Eight 5-week-old *Gt(ROSA)26Sor*^*Cre/Cre*^*Apoc2*^*fl/fl*^ mice from the same litter showed comparable body weight increase 3 weeks post-injection (2 M + 2 F injected with TAM, 2 M + 2 F injected with corn oil) (experiment 8). Shapiro–Wilk test was used to test the normality of data, and unpaired two-tailed *t*-test was used to compare the groups (*P* = 0.631). **c**, Pictures of spleens and livers collected from 2 female *Apoc2-*KO mice 5 months after TAM injection and two female WT mice (*Gt(ROSA)26Sor*^*wt/wt*^*Apoc2*^*fl/fl*^) 4 months after TAM injection from experiment 7. **d**–**g**, Mice came from experiments 1, 2 and 5–7 in Supplementary Table 1. The KO group included five male and five female mice, while the WT group included one male CD45.2^+^ mouse injected with TAM and seven *Gt(ROSA)26Sor*^*wt/wt*^*Apoc2*^*fl/fl*^ mice (*n* = 3 M + 4 F) injected with TAM. Waiting time after injection in the *Apoc2-*KO group varied from 11 days to 5 months, and 11 days to 4 months in the WT group. The weights of liver tissues (**d**) and spleen tissues (**e**) were compared between two groups. The bar graph represents the mean of tissue mass in *Apoc2-*KO mice (*n* = 10) and WT (*n* = 8) with s.d. Each colored dot represents the tissue mass value of every single mouse tissue. Shapiro–Wilk test was used to test the normality of data and unpaired two-tailed *t*-test was used to compare groups. The liver (**f**) and spleen (**g**) tissue mass was normalized to the age of the mouse and then compared between the groups. The bar graph shows the mean tissue mass normalized to the age of each mouse in the WT and *Apoc2*-KO groups, with s.d. Shapiro–Wilk test was used to assess data normality and unpaired *t*-test was used to analyze differences in liver tissue, while Mann–Whitney test was applied to spleen tissue, as the spleen data were not normally distributed (**g**) (*P < 0.05; n.s., not significant). Data are presented as mean ± s.d., and each dot represents a mouse. Source data

Body weights of the *Apoc2-*KO and WT groups that received TAM or corn oil injections were recorded. No significant difference in body weight was observed between the *Apoc2-*KO (*n* = 26) and WT (*n* = 22) groups (experiments 1, 5, 6 and 8 and live mice that were injected with TAM but not included in any experiment) (23.44 g versus 26.42 g, *P* = 0.173; Fig. 2a). Age-normalized weight was also comparable between groups (Supplementary Fig. 3a). In addition, eight 5-week-old *Gt(ROSA)26Sor*^*Cre/Cre*^*Apoc2*^*fl/fl*^ mice from the same litter were randomized into two groups (2 M + 2 F per group). One group was injected with TAM (*Apoc2*-KO), and another group was injected with corn oil (WT control) (experiment 8). *Apoc2-*KO and WT mice (2.45 g versus 2.13 g, *P* = 0.631; Fig. 2b) showed comparable increases in body weight 3 weeks post-final injection. Liver and spleen tissues of *Apoc2-*KO mice appeared normal in shape and color and looked similar to those from WT mice (experiment 7) (Fig. 2c and Supplementary Fig. 3b). The average liver weight of *Apoc2-*KO mice was slightly lower than but not statistically significantly different from that of the WT mice (1.60 g versus 1.95 g, *P* = 0.172). When liver weights were normalized by age, no significant differences were observed (*Apoc2*-KO versus WT: 0.12 versus 0.10, *P* = 0.469). Spleen weights of *Apoc2-*KO mice were significantly lower than those in WT mice (0.11 g versus 0.13 g, *P* = 0.041). However, age-normalized spleen weights showed no significant differences between the groups (*Apoc2-*KO versus WT: 0.0078 versus 0.0070, *P* = 0.762). These data are shown in Fig. 2d–g.

### *Apoc2-*KO mice exhibit elevated triglyceride levels

**Fig. 3:**
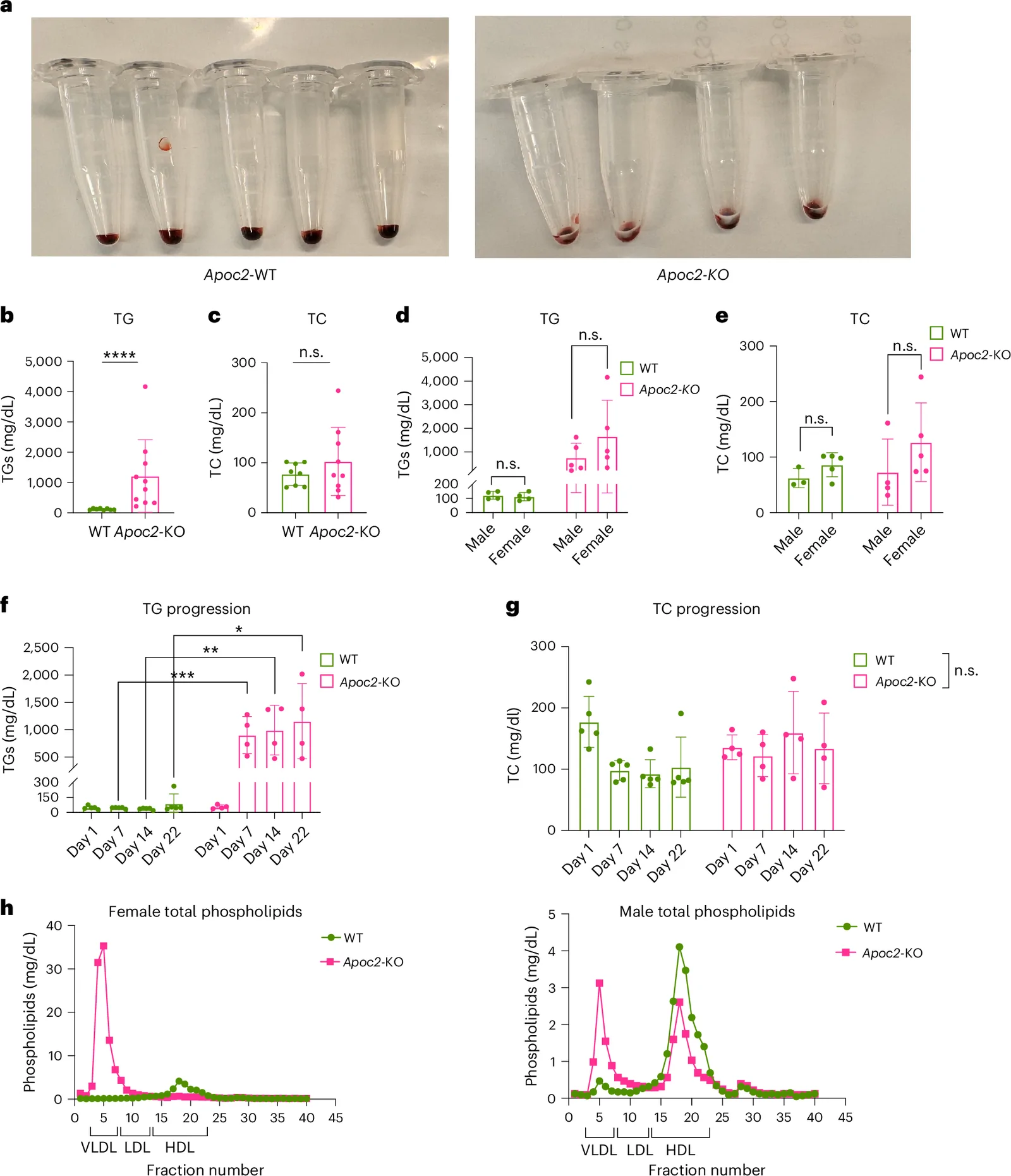
*Apoc2-*KO mice showed higher concentrations of TGs in serum. **a**, Representative blood pictures collected on day 14 after TAM injection from *Apoc2-*KO mice (*n* = 2 M + 2 F) and WT mice (*n* = 2 M + 3 F) (experiment 10). Blood samples were kept at room temperature for 30 min in EDTA-free tubes to allow clot formation. Pictures were taken right after the centrifugation for serum collection. **b**,**c**, Serum TG (**b**) and TC (**c**) concentrations were measured using a colorimetric assay (*Apoc2-*KO: *n* = 10 (5 M + 5 F) for TG, *n* = 9 (4 M + 5 F) for TC; WT: *n* = 8 for both assays). WT mice included two male CD45.2^+^, six *Gt(ROSA)26Sor*^*wt/wt*^*Apoc2*^*fl/fl*^ (*n* = 2 M + 4 F) in TG assay and two male CD45.2^+^, six *Gt(ROSA)26Sor*^*wt/wt*^*Apoc2*^*fl/fl*^ (*n* = 1 M + 5 F) in TC assay. Serum samples were collected at 11 days, 1 month or 5 months (experiments 1, 4, 7 and 14). All WT mice were injected with TAM for 5 days. Each colored dot represents an individual serum sample from one mouse. Shapiro–Wilk test was used to test the normality of data. Differences in TG levels between *Apoc2-*KO and WT groups were analyzed by Mann–Whitney test (*P* < 0.0001), while TC levels were analyzed using an unpaired two-tailed *t*-test (*P* = 0.329). **d**,**e**, Sex-based comparisons of TG (**d**) and TC (**e**) levels in *Apoc2-*KO and WT groups. TG analysis included *Apoc2-*KO mice (*n* = 5 M + 5 F), and WT mice (*n* = 4 M + 4 F). TC analysis included *Apoc2-*KO mice (*n* = 4 M + 5 F) and the WT mice (*n* = 3 M + 5 F). Each dot represents an individual serum sample. The differences between males and females within each group were analyzed using a two-tailed unpaired *t*-test. **f**,**g**, 2 M + 2 F *Apoc2*-KO mice and 2 M + 3 F WT mice (*Gt(ROSA)26Sor*^*wt/wt*^*Apoc2*^*fl/fl*^ injected with TAM) were monitored for TG (**f**) and TC (**g**) progression assay over 22 days (experiment 10; Supplementary Table 1). Each colored dot represents an individual serum sample from one mouse. The statistical difference between two groups on the same day was analyzed by a multiple unpaired *t*-test. **h**, Total phospholipid profiles in female and male mice were assessed separately (*n* = 2 M + 2 F each group) (experiment 11). (****P < 0.0001; ***P < 0.001; **P < 0.01; *P < 0.05). Data are presented as mean ± s.d., and each dot represents a mouse. Source data

Serum samples were collected 11 days, 1 month or 5 months after TAM injection (experiments 1, 4, 7 and 14), and serum triglycerides (TGs) and total cholesterol (TC) levels were measured using a colorimetric assay. Notably, TG variability was high in the *Apoc2-*KO group, and several *Apoc2-*KO mice exhibited milky serum (Fig. 3a). Overall, serum from *Apoc2-*KO mice exhibited significantly elevated TG levels compared with WT mice (1,212.9 mg/dL versus 118.1 mg/dL, *P* < 0.0001; Fig. 3b). Although TC levels were elevated in some *Apoc2*-KO mice, the overall difference between *Apoc2*-KO and WT mice was not statistically significant (102.9 mg/dL versus 77.39 mg/dL, *P* = 0.329; Fig. 3c). No significant differences in TG levels were observed between male and female mice within each group (*Apoc2-*KO: male versus female—759.0 mg/dL versus 1,667 mg/dL, *P* = 0.253; WT: male versus female—122.9 mg/dL versus 113.3 mg/dL, *P* = 0.649; Fig. 3d) or TC levels (*Apoc2-*KO: male versus female—72.95 mg/dL versus 126.9 mg/dL, *P* = 0.264; WT: male versus female—62.51 mg/dL versus 86.31 mg/dL, *P* = 0.160; Fig. 3e). The progression of TG and TC levels was also examined and compared between four *Apoc2*-KO mice and five WT mice on days 1, 7, 14 and 22 after TAM injection (experiment 10; Fig. 3f,g). Starting from day 7, the TG levels of *Apoc2*-KO mice were significantly higher than those of WT mice (*Apoc2*-KO versus WT: 903.8 mg/dL versus 44.93 mg/dL on day 7, *P* = 0.0007; 995.1 mg/dL versus 35.84 mg/dL on day 14, *P* = 0.002; 1,157.39 mg/dL versus 88.80 mg/dL on day 22, *P* = 0.01) and showed continuous increase. However, TC levels and progression were not significantly different between the two groups (*Apoc2*-KO versus WT: 134.0 mg/dL versus 103.4 mg/dL on day 22, *P* = 0.416).

To assess whether the significant elevation of TG levels in *Apoc2*-KO mice may impact plasma VLDL and HDL, plasma phospholipids profiles were determined by a fast protein liquid chromatography (FPLC) system with pooled mouse plasma (*n* = 2 M + 2 F each group (experiment 11)). Female *Apoc2*-KO mice showed clearly decreased amount of HDL compared with WT mice, while this effect was less pronounced in *Apoc2*-KO males. Both female and male *Apoc2*-KO mice showed higher VLDL than WT mice, and similar low-density lipoprotein (LDL) levels were observed between the two groups (Fig. 3h).

### *Apoc2* deletion did not impact the in vitro expansion of BM cells

**Fig. 4:**
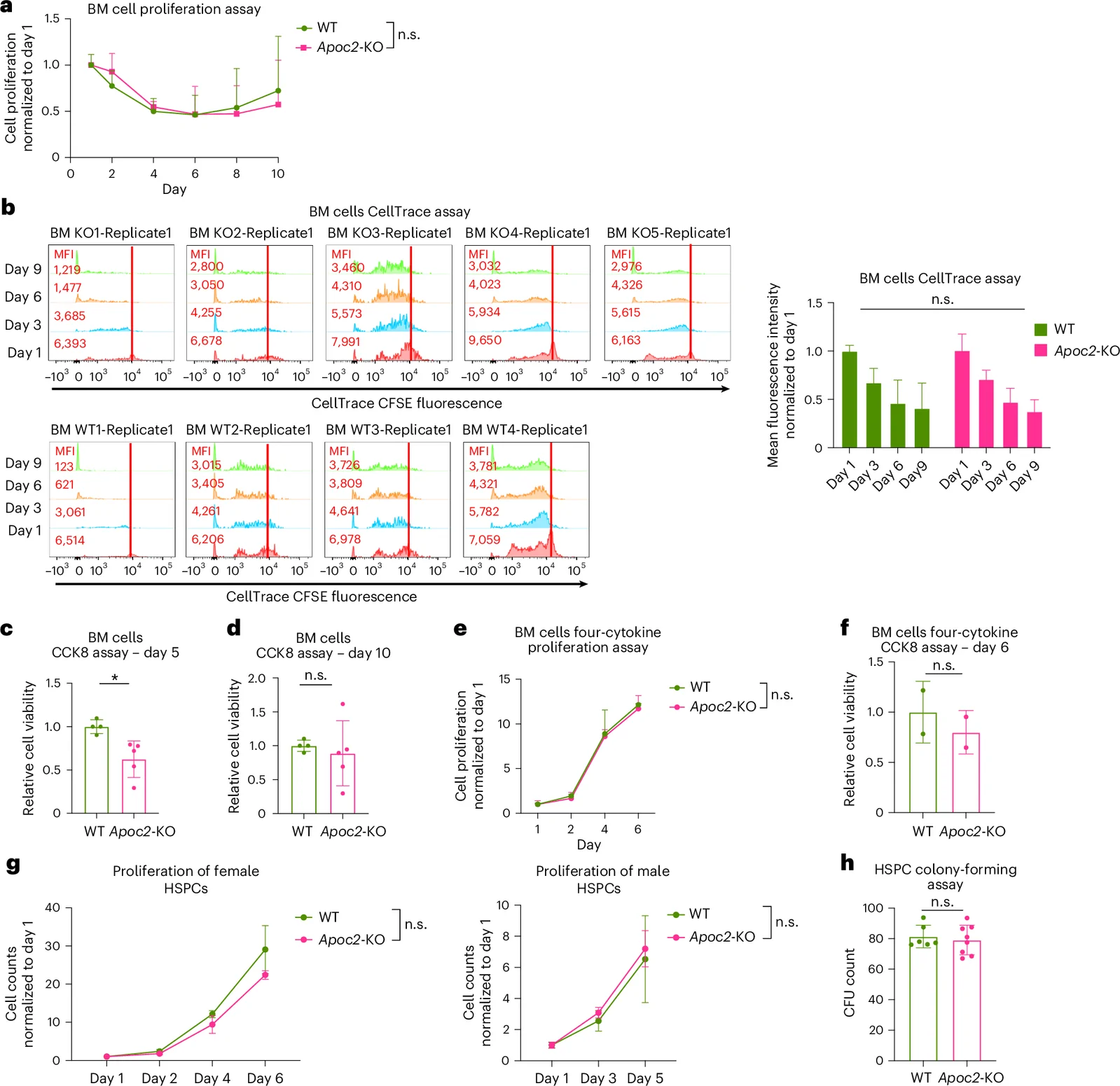
*Apoc2* deletion did not significantly impact the in vitro expansion of BM cells. **a**, A 10-day trypan blue cell count assay was conducted. Each mouse had two technical replicates (*n* = 5 mice in *Apoc2*-KO group, 4 in WT group). Waiting time after TAM injections was: 11 days (1 male CD45.2^+^ mouse in WT group, 1 male *Apoc2*-KO mouse), 6 weeks (2 female *Gt(ROSA)26Sor*^*wt/wt*^*Apoc2*^*fl/fl*^ mice in WT group, 2 females in *Apoc2*-KO group) and 1 male *Gt(ROSA)26Sor*^*wt/wt*^ *Apoc2*^*fl/fl*^ mouse and 1 male *Apoc2*-KO mouse received 2 rounds of TAM injections (5 days per round) with a 3-week interval between two rounds and 1-week waiting time after the second round (experiments 1, 2 and 5). BM cell numbers from both groups were normalized to day 1 values and compared daily. Shapiro–Wilk test was used to test the normality of data. Statistical analysis was performed by multiple unpaired *t*-test. **b**, A 9-day CellTrace assay was conducted on the same mice. Each mouse had two technical replicates (*n* = 5 mice in *Apoc2*-KO group, 4 in WT group). Flow histograms for the first replicate of each mouse are shown with MFI values. MFI values were normalized to day 1 and compared between groups. Shapiro–Wilk test was used to test the normality of the data. A multiple unpaired *t*-test was performed to compare the normalized MFI values between two groups within the same day. **c**,**d**, CCK8 assays were performed on day 5 (**c**) and day 10 (**d**) with 4 technical replicates per mouse (*n* = 5 mice for *Apoc2-*KO, *n* = 4 for WT). OD values were normalized to the average OD of the WT group to determine relative cell viability. Shapiro–Wilk test was used for normality testing and unpaired *t*-test for group comparisons. **e**,**f**, BM cells were cultured in R20 supplemented with SCF, TPO, IL-3 and IL-6 cytokines (*n* = 2 mice per group). Mice were euthanized 6 months after TAM injection. WT mice were CD45.2^+^ males injected with TAM (experiment 9). The trypan blue cell counting assay (**e**) was set up with two technical replicates per mouse. Cells were counted on days 1, 2, 4 and 6. Shapiro–Wilk test was used to test the normality of the data. Statistical analysis was performed using a multiple unpaired *t*-test. The CCK8 assay (**f**) was measured on day 6 with six technical replicates per mouse. OD values were normalized to the average OD of the WT group to determine relative cell viability. Shapiro–Wilk test was used to test the normality of the data. Statistical analysis was performed using an unpaired *t*-test. **g**, HSPCs were isolated from BM from two CD45.2^+^ WT mice injected with TAM and two *Apoc2*-KO mice (*n* = 1 M + 1 F per group) about 1 year post-injection (10–15 months) (experiment 12). Proliferation was performed for 6 days. Cell counts were normalized to the count on day 1. Statistical analysis was performed using a multiple unpaired *t*-test. **h**, Colony-forming assay was performed with BM cells from the same mice used in proliferation assay, and an additional 3 *Apoc2*-KO males and 2 *Gt(ROSA)26Sor*^*wt/wt*^*Apoc2*^*fl/fl*^ males euthanized 3 months after TAM injections were included in the assay (experiment 6; Supplementary Table 1). Colony-forming units were manually counted for each replicate. Each mouse had two technical replicates (*n* = 5 M + 3 F mice in *Apoc2*-KO group, 4 M + 2 F in WT group). Data represent the mean colony counts with s.d. Each dot represents the average count for an individual mouse. Shapiro–Wilk test was used to assess normality, and the Mann–Whitney test was used for group comparisons (*P < 0.05). Source data

BM cells obtained from mice of experiments 1, 2 and 5 (Supplementary Table 1) were included to assess their in vitro proliferation potential. Cells were cultured in RPMI medium supplemented with 20% fetal bovine serum (R20) and stem cell factor (SCF) and thrombopoietin (TPO) cytokines for hematopoietic stem and progenitor cell (HSPC) expansion for 10 days in vitro. BM cells obtained from both WT mice (*n* = 2 M + 2 F) and *Apoc2-*KO mice (*n* = 2 M + 3 F) (experiments 1, 2 and 5) started to expand on the eighth day of in vitro culture (Fig. 4a). Daily count of viable cells showed no significant difference between the BM cells from *Apoc2-*KO and WT mice (*P* > 0.05). Similarly, the 9-day CellTrace assay showed that the mean fluorescence intensity (MFI) of *Apoc2-*KO BM cells on day 9 normalized to the first day was slightly lower but not statistically significant than that of WT group (*Apoc2-*KO versus WT: 0.37 versus 0.41, *P* = 0.802; Fig. 4b and Supplementary Fig. 4). However, Cell Counting Kit-8 (CCK8) assay measuring cellular dehydrogenases in metabolically active cells showed lower levels in *Apoc2-*KO BM cells compared with WT cells on day 5 (*P* = 0.012; Fig. 4c), but not on day 10 (*P* = 0.665; Fig. 4d). Because BM cells cultured with SCF and TPO showed limited in vitro expansion, we cultured BM cells (experiment 9) in media supplemented with SCF, TPO, interleukin (IL)-3 and IL-6 cytokines and assessed their proliferation over six days. BM cells were expanded in vitro, and no significant difference between groups was observed (Fig. 4e). Relative cell viability measured by CCK8 was also similar between groups on day 6 (*P* = 0.529; Fig. 4f). The proliferation assay was also performed with isolated HSPCs (*n* = 1 M + 1 F per group (experiment 12)) to test the effects of *Apoc2* KO on HSPC expansion. WT and *Apoc2*-KO HSPCs showed similar proliferation trends (Fig. 4g). Similarly, in two male mice (experiment 12) receiving 5-fluorouracil injection 4 days before euthanasia, the HSPCs from *Apoc2*-KO male mouse showed similar proliferation to that of WT HSPCs. To investigate whether *Apoc2* KO impacts the colony-forming ability of hematopoietic stem cells, BM cells from WT and *Apoc2-*KO mice (Supplementary Table 1, experiments 1, 2, 5 and 6) were cultured in a semi-solid cytokine-supplemented methylcellulose based medium. The colonies counted on day 7 after plating showed a similar number of colonies between the groups (*Apoc2-*KO versus WT: 79.09 versus 81.47 colonies, *P* = 0.687; Fig. 4h).

### Deletion of *Apoc2* showed minimal impact on the hematological profile

**Fig. 5:**
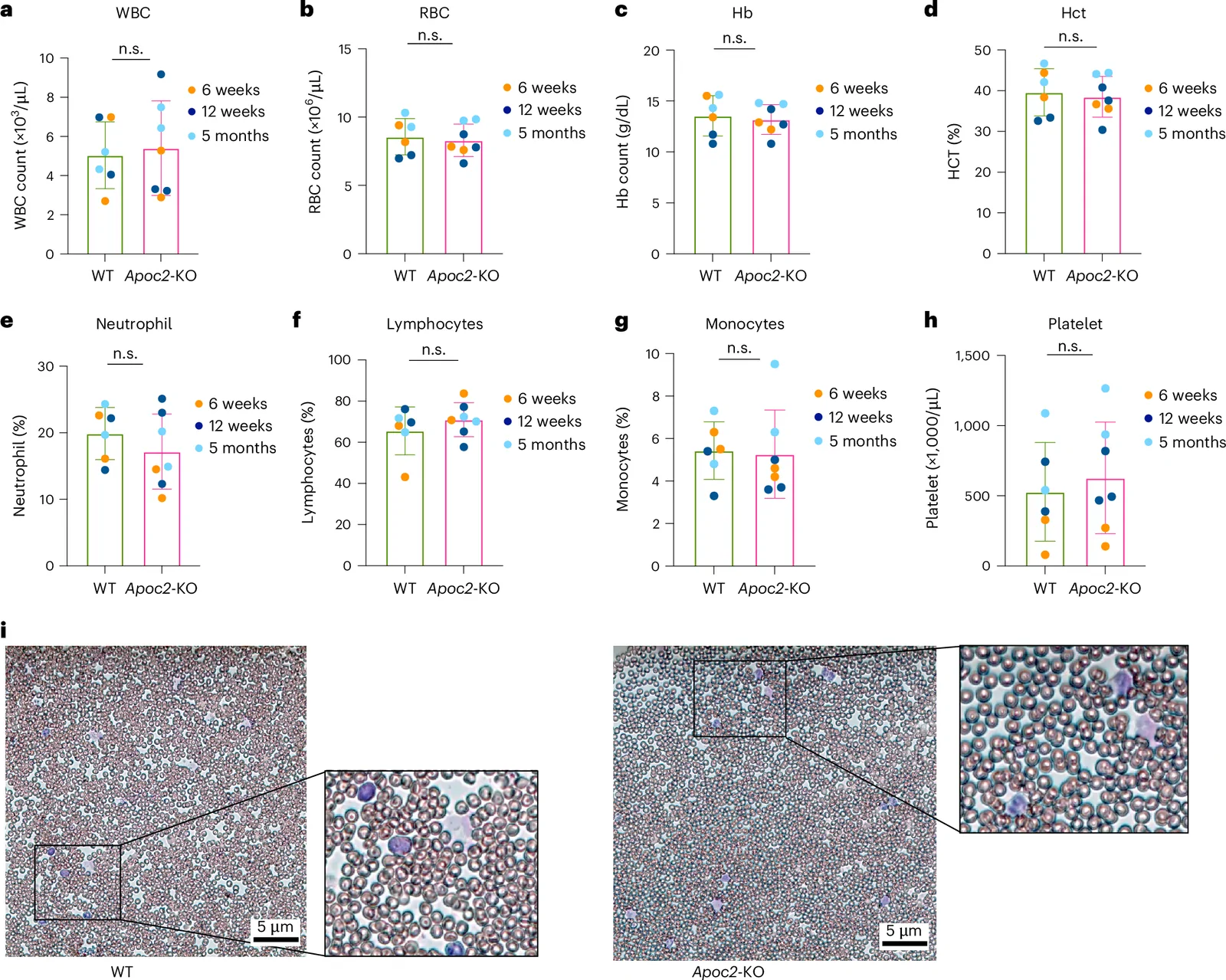
Morphological assessments revealed comparable blood phenotypes between *Apoc2*-KO and WT mice. **a**–**h**, The post-TAM injection waiting time before collecting heart blood from mice included 6 weeks (2 females in *Apoc2*-KO group, 2 *Gt(ROSA)26Sor*^*wt/wt*^*Apoc2*^*fl/fl*^ females in WT group), 12 weeks (3 males in *Apoc2*-KO group, 2 *Gt(ROSA)26Sor*^*wt/wt*^*Apoc2*^*fl/fl*^ males in WT group) and 5 months (2 females in *Apoc2*-KO group, 2 *Gt(ROSA)26Sor*^*wt/wt*^*Apoc2*^*fl/fl*^ females in WT group). Bar graphs show the mean ± s.d. of blood counts for *Apoc2*-KO mice (*n* = 7) and WT mice (*n* = 6) for WBCs (**a**), RBCs (**b**), Hb (**c**), HCT (%) (**d**), neutrophils (%) (**e**), lymphocytes (%) (**f**), monocytes (%) (**g**) and platelets (**h**). Each dot represents an individual mouse: orange dots are from experiment 5, dark-blue dots from experiment 6 and sky-blue dots from experiment 7. The Shapiro–Wilk test was used to assess normality, and an unpaired two-tailed *t*-test was used for group comparisons. **i**, Fresh tail blood smear of WT and *Apoc2*-KO male mice (*n* = 4 per group) were examined by Wright–Giemsa staining under 20× Zeiss Scope A1 microscope. One representative picture was selected from each group. Source data

To evaluate the impact of *Apoc2* KO on mouse hematological profiles, complete blood counts were performed on blood samples from *Apoc2-*KO mice (3 M + 4 F) and compared with WT mice (2 M + 4 F) 6 weeks, 3 months or 5 months after TAM injection (experiments 5–7). Hematological parameters including white blood cell (WBC) count, red blood cell (RBC) count, hemoglobin (Hb), hematocrit (HCT), mean corpuscular volume (MCV), mean corpuscular Hb (MCH), MCH concentration (MCHC), neutrophils, lymphocytes, monocytes, eosinophils, basophils and platelet counts are summarized in Supplementary Table 2. Overall, the comparison showed no significant differences in WBC, RBC, Hb or lymphocyte percentages between the groups (Fig. 5a–h), indicating minimal effects of *Apoc2* KO on mouse hematological profiles.

To assess potential subtle changes in cellular differentiation or dysplasia, Wright–Giemsa staining was done with WT and *Apoc2*-KO peripheral blood (*n* = 4 males per group) (Supplementary Fig. 5). Two of *Apoc2*-KO mice were injected with TAM at 2 months of age and two were injected TAM at 6 months of age. In WT control group, all mice were *Gt(ROSA)26Sor*^*Cre/Cre*^*Apoc2*^*wt/wt*^ males. Two of them were injected with TAM at 1 month of age, and the other two were injected with TAM at 2 months of age (experiment 13). Blood samples were collected around 1.5 years later. Histological analysis did not reveal notable differences in cell morphologies between *Apoc2*-KO and WT mice, including no evidence of dysplasia or abnormal differentiation in blood cells, suggesting limited long-term impact of *Apoc2* deletion on mature blood cell morphology (Fig. 5i and Supplementary Fig. 6).

### *Apoc2* deletion did not impact the in vitro expansion of T cells

**Fig. 6:**
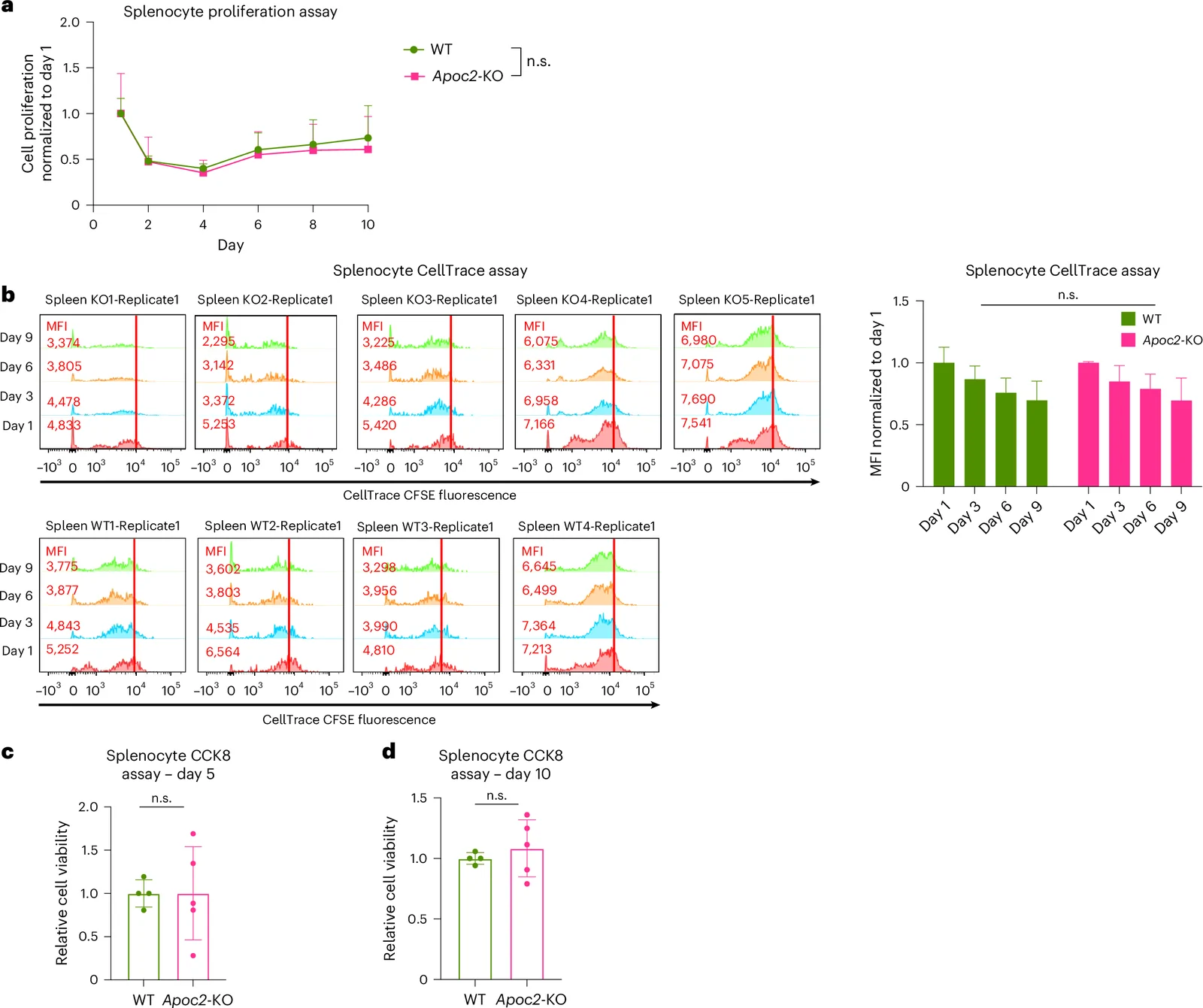
*Apoc2* deletion did not affect the in vitro expansion of T cells. **a**, A 10-day trypan blue cell count assay was conducted on mouse primary splenocytes with two technical replicates per mouse (*n* = 2 M + 3 F mice for the *Apoc2*-KO group, *n* = 2 M + 2 F for the WT group). Cell counts for both groups were normalized to day 1 and compared daily using a multiple unpaired *t*-test. **b**, A 9-day CellTrace assay was performed on mouse primary splenocytes with 2 technical replicates per mouse (*n* = 5 mice for the *Apoc2*-KO group, *n* = 4 mice for the WT group), on the same day as the plating for the cell proliferation assay. Flow histograms of the first replicate for each mouse are shown with MFI values. MFI values for both groups were normalized to day 1 and compared daily using a multiple unpaired *t*-test. **c**,**d**, CCK8 assays were conducted on day 5 (**c**) and day 10 (**d**) (*n* = 5 mice in the *Apoc2*-KO group, *n* = 4 mice in the WT group). OD values for each mouse were normalized to the average OD values of the WT group to calculate relative cell viability. The Shapiro–Wilk test was used to assess normality, and an unpaired two-tailed *t*-test was used for group comparisons. Source data

To evaluate the impact of *Apoc2* KO on the in vitro expansion of T cells, mouse splenocytes were cultured in R20 medium supplemented with IL-2 and phytohemagglutinin (PHA) to support T cell proliferation. Daily trypan blue cell counting over 10 days revealed that T cells from both groups (experiments 1, 2 and 5) began proliferating on day 6, with no significant differences observed between *Apoc2*-KO and WT groups on any day (*P* > 0.05; Fig. 6a). Similar findings were confirmed using the CellTrace assay (*P* > 0.05; Fig. 6b and Supplementary Fig. 6). Furthermore, the CCK8 assay showed no significant differences in the number of metabolically active cells between *Apoc2*-KO and WT splenocytes on days 5 and 10 (Fig. 6c,d).

### *Apoc2* deletion had a slight impact on competitive BM repopulation capability

**Fig. 7:**
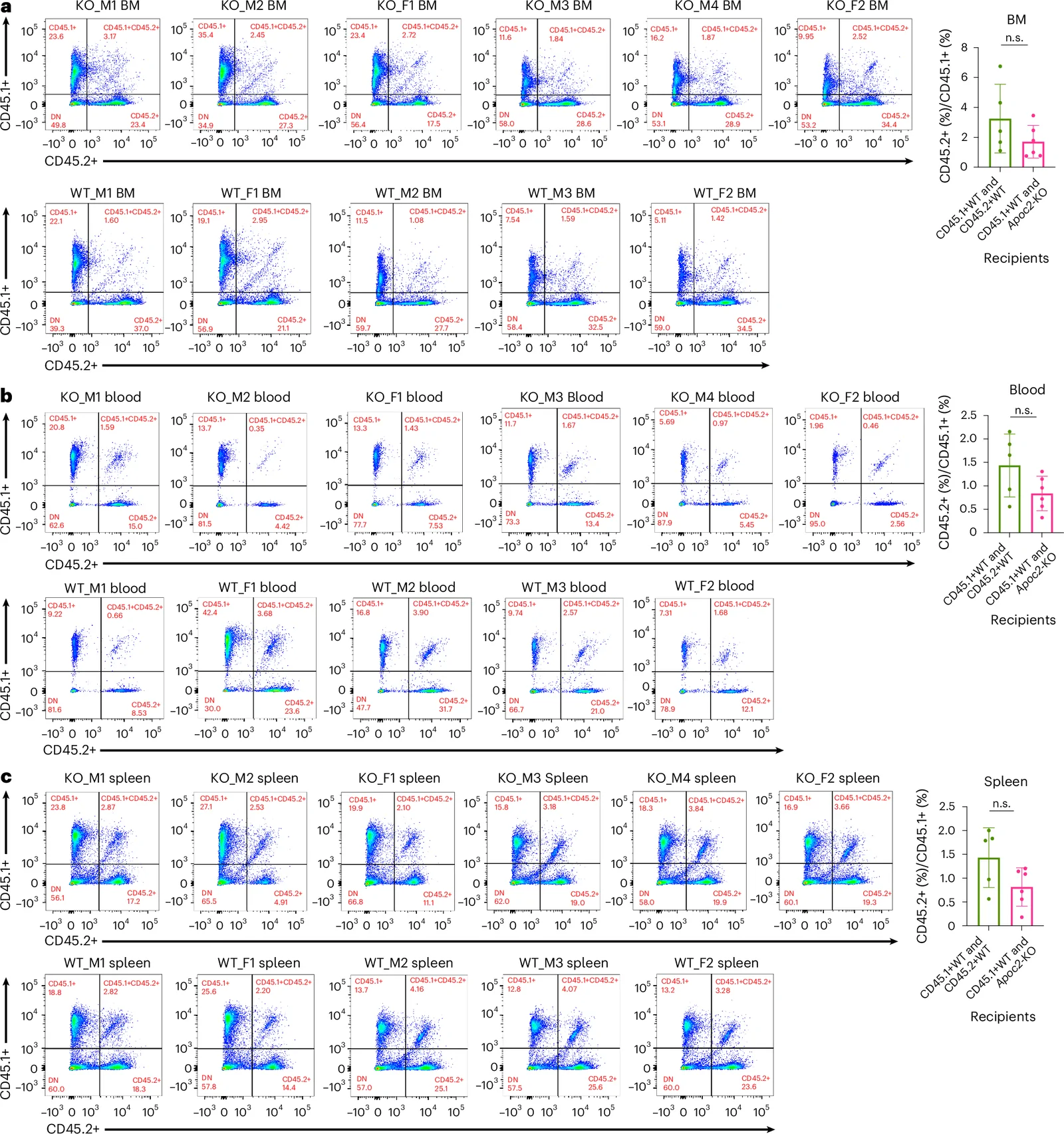
*Apoc2* deletion did not significantly reduce competitive BM repopulation ability. **a**–**c**, Flow cytometry results show the engraftment of *Apoc2*-KO BM cells, CD45.2^+^ WT BM cells, and CD45.1^+^ WT BM cells in the BM (**a**), the blood (**b**) and the spleen (**c**) tissues of CD45.1^+^CD45.2^+^ irradiated recipient mice. Approximately 7 × 10^6^ CD45.2^+^ *Apoc2*-KO BM cells were mixed with an equal number of CD45.1^+^ WT BM cells and injected intravenously into irradiated CD45.1^+^CD45.2^+^ recipient mice (*n* = 4 M + 2 F). Similarly, 7 × 10^6^ CD45.2^+^ WT BM cells were mixed with an equal number of CD45.1^+^ WT BM cells and injected into irradiated recipients (*n* = 3 M + 2 F). Each dot represents the engraftment percentage of donor cells in individual recipient mice. Quantification of engraftment percentages of donor cells in the BM, blood and spleen tissues by flow cytometry were analyzed using an unpaired two-tailed *t*-test. Source data

To examine whether *Apoc2* KO may affect the repopulation ability of HSPCs, *Apoc2-*KO BM cells (CD45.2^+^) were mixed with CD45.1^+^ WT BM cells at a 1:1 ratio to be transplanted into CD45.1^+^CD45.2^+^ irradiated mice (*n* = 2 M + 1 F). To determine whether the difference between the groups was caused by the KO of *Apoc2* or the inherently different repopulation ability between the CD45.1^+^ and CD45.2^+^ backgrounds, the same number of BM cells from CD45.1^+^ WT donors and CD45.2^+^ WT donors were mixed at a 1:1 ratio to be transplanted into one male and one female CD45.1^+^CD45.2^+^ irradiated recipients. CD45.1 and CD45.2 monoclonal antibodies were used to distinguish single CD45.2^+^ (*Apoc2*-KO or WT donor), single CD45.1^+^ (competitive donor) and double CD45.1^+^CD45.2^+^ (recipient) cells. On day 34 after transplantation, all recipient mice were euthanized. The competitive repopulation assay was repeated with the same number of donor cells and the same waiting time (*n* = 2 M + 1 F per recipient group). Liver and spleen tissues showed comparable morphology and weights between mice engrafted with WT and *Apoc2*-KO cells (Supplementary Fig. 7). In the first experiment (Supplementary Table 1, experiment 3), donor BM cells came from CD45.2^+^ *Apoc2-*KO mice, CD45.2^+^ WT mice and CD45.1^+^ WT mice (*n* = 2 M per genotype); all mice were euthanized 1 month after TAM injection. In the repeated experiment (Supplementary Table 1, experiment 4), donors included CD45.2^+^ *Apoc2-*KO mice, *Gt(ROSA)26Sor*^*wt/wt*^*Apoc2*^*fl/fl*^ mice (CD45.2^+^) that had received TAM and CD45.1^+^ WT mice (*n* = 1 M + 1 F per genotype). The waiting time after TAM injection was also 1 month. The reconstitution in BM, spleen and blood cells was measured by flow cytometry. In recipients’ BM, the mean percentage of CD45.2^+^ WT cells was 17.5% higher than that of CD45.1^+^ WT cells (*P* = 0.0037), and CD45.2^+^ *Apoc2-*KO cells were 6.7% higher than CD45.1^+^ WT cells (*P* = 0.171). In both blood and spleen tissues, the mean percentage of CD45.2^+^ *Apoc2-*KO cells was lower than that of CD45.1^+^ WT cells, but with no significant difference. CD45.2^+^ WT cells exhibited higher engraftment than CD45.1^+^ WT cells, but the result was not significant. Combining data from the two experiments revealed that the mean ratio of CD45.2^+^ *Apoc2-*KO cells to CD45.1^+^ WT cells was lower than that of CD45.2^+^ WT cells to CD45.1^+^ WT cells; however, the difference was not statistically significant. In the BM, the mean ratio of CD45.2^+^ *Apoc2-*KO/CD45.1^+^ WT was lower than that of the CD45.2^+^ WT/CD45.1^+^ WT (*Apoc2-*KO/CD45.1^+^ WT versus CD45.2^+^ WT/CD45.1^+^ WT: 1.70 versus 3.25, *P* = 0.175; Fig. 7a). The mean ratio of CD45.2^+^ to CD45.1^+^ in recipient’s blood was also lower for *Apoc2-*KO compared with *Apoc2-*WT mice (*Apoc2-*KO/CD45.1^+^ WT versus CD45.2^+^ WT/CD45.1^+^ WT: 0.84 versus 1.44, *P* = 0.092; Fig. 7b). Similar trends were observed in recipients’ spleen tissue (*Apoc2-*KO/CD45.1^+^ WT versus CD45.2^+^ WT/ CD45.1^+^ WT: 0.82 versus 1.43, *P* = 0.080; Fig. 7c). Flow cytometry gating strategies are shown in Supplementary Fig. 8.

### RNA-sequencing revealed the enrichment of heme metabolism- and lipid metabolism-related pathways in *Apoc2-*KO BM cells

**Fig. 8:**
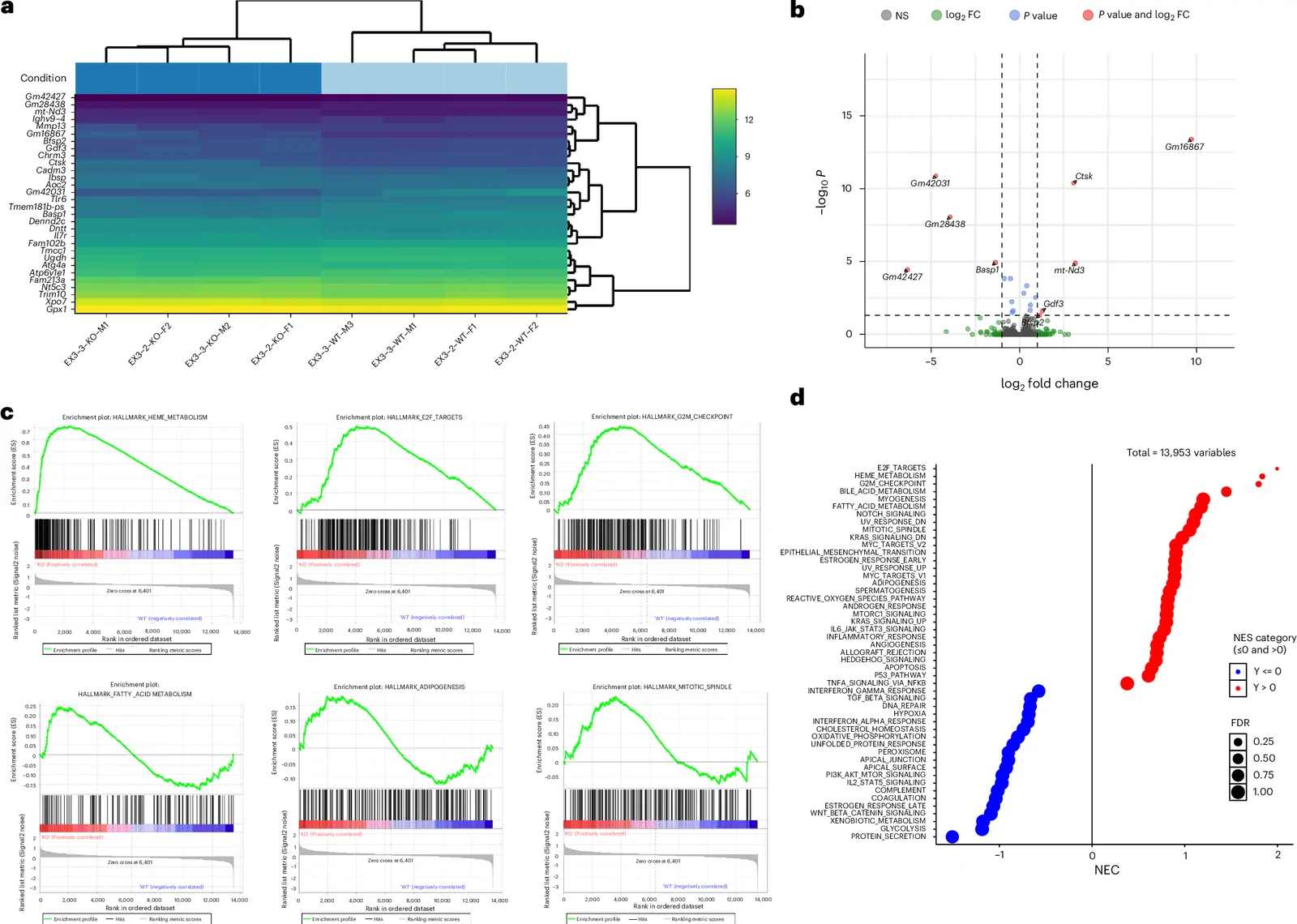
RNA-seq analysis revealed the differential gene expression in *Apoc2-*KO mice in comparison with WT mice. **a**, A biclustering heatmap showing the top 30 differentially expressed genes sorted by the adjusted *P* value by plotting their log_2_-transformed expression values in samples. **b**, Volcano plot showing the log_2_ fold change and the significance of gene expression difference between the *Apoc2*-KO group and the WT group. Genes with an adjusted *P* value less than 0.05 and a log_2_ fold change greater than 1 or less than −1 are indicated by red dots. The names of five upregulated genes (*Gm16867*, *mt-Nd3*, *Ctsk*, *Gdf3* and *Bfsp2*) and four downregulated genes (*Gm42427*, *Gm42031*, *Gm28438* and *Basp1*) are shown. **c**, Enrichment plots generated by GSEA. **d**, Pathway analysis identified the differentially expressed pathways categorized by NES along with the FDR. The Wald test was used to calculate *P* values and log_2_ fold changes. Genes with the adjusted *P* value <0.05 and absolute log_2_ fold change >1 were identified as differentially expressed genes. FDR below 25% was considered significant. Source data

RNA sequencing (RNA-seq) of BM RNA samples from four female and four male mice was performed to identify differentially expressed genes between WT and *Apoc2*-KO mice (*n* = 2 F + 2 M per group). All WT mice were *Gt(ROSA)26Sor*^*wt/wt*^*Apoc2*^*fl/fl*^ mice that received TAM injections. The waiting time after TAM injection was 6 weeks for all females and 3 months for males (experiments 5 and 6). The top 30 differentially expressed genes, ranked by adjusted *P* value, were visualized using a biclustering heatmap (Fig. 8a) and a volcano plot (Fig. 8b). In the *Apoc2*-KO group, five upregulated (*Gm16867*, *mt-Nd3*, *Ctsk*, *Gdf3* and *Bfsp2*) and four downregulated genes (*Gm42427*, *Gm42031*, *Gm28438* and *Basp1*) were identified compared with the WT group. Among these, the significantly upregulated *mt-Nd3* (log_2_ fold change 3.17, *P* < 0.0001, *q* < 0.0001) encodes a protein orthologous to human *MT-ND3*, which facilitates NADH dehydrogenase activity in the electron transport chain for ATP production^15^. *Ctsk* (log_2_ fold change 3.07, *P* < 0.0001, *q* < 0.0001) is related to osteoclast-mediated bone destruction^16^. *Gdf3* (log_2_ fold change 1.3, *P* < 0.0001, *q* = 0.03), a gene that is involved in early development and most prominently expressed in adult adipose tissue^17–19^, and *Bfsp2* (log_2_ fold change 1.14, *P* < 0.0001, *q* = 0.05), a gene involved in sustaining lens cell morphology^20^, were also upregulated in *Apoc2-*KO BM. *Gm16867* gene (log_2_ fold change 9.69, *P* < 0.0001, *q* < 0.0001) was upregulated in the BM of *Apoc2-*KO mice (Fig. 8a,b). *Gm16867* is orthologous to human solute carrier family 25 member 37 (*SLC25A37*). Solute carrier family 25 (SLC25) is the largest solute transporter family in humans^21^. Mitoferrin-1 encoded by *SLC25A37*^22^ is localized in the mitochondrial inner membrane and acts as an essential iron importer for the synthesis of mitochondrial heme and iron–sulfur clusters in erythroblasts^23^, controlling the balance of iron in the cytoplasm and mitochondria^22^. *Basp1* (log_2_ fold change −1.34, *P* < 0.0001, *q* < 0.0001; Fig. 8a,b) was also downregulated in *Apoc2-*KO BM. The human *BASP1* gene has been reported to have a tumor-suppressive effect in many cancers^24^. Other downregulated genes in the *Apoc2-*KO BM included the pseudogene *Gm28438* (log_2_ fold change −3.90, *P* < 0.0001, *q* < 0.0001), noncoding RNA gene *Gm42031* (log_2_ fold change −4.70, *P* < 0.0001, *q* < 0.0001) and the protein-coding gene *Gm42427* (log_2_ fold change −6.30, *P* < 0.0001, *q* < 0.0001) (Fig. 8a,b).

Gene set enrichment analysis (GSEA) revealed a significant enrichment of the cell cycle-related pathways, E2F targets (normalized enrichment score (NES) 2.00, *P* = 0.025, *q* = 0.031) and G2M checkpoint (NES 1.80, *P* = 0.058, *q* = 0.090) in the *Apoc2-*KO group (Fig. 8c,d). Heme metabolism pathway (NES 1.84, *P* < 0.0001, *q* = 0.077) was also found to be upregulated in the BM of *Apoc2-*KO mice (Fig. 8c,d). Pathways relevant to lipid synthesis and metabolism, such as fatty acid metabolism (NES 1.18, *P* = 0.133, *q* = 0.968), adipogenesis (NES 0.87, *P* = 0.645, *q* = 1.000) and mTORC1 signaling (NES 0.80, *P* = 0.741, *q* = 1.000) were positively enriched in the *Apoc2-*KO group*;* however, the results were not statistically significant (Fig. 8c,d).

## Discussion

We have previously found that *APOC2* was upregulated in AML^3^. The knockdown of *APOC2* reduced leukemia growth in vitro and decreased the leukemia burden in a human AML xenograft mouse model. We also found that APOC2 cooperates with CD36 to activate the ERK signaling pathway and promote the leukemia growth^3^. Our studies revealed APOC2 and CD36 as potential therapeutic targets in AML. To develop novel therapeutic approaches against APOC2/CD36, understanding their role in normal cells is critical. We have recently assessed the role of CD36 deletion in normal hematopoiesis and found limited overall detrimental impact of Cd36 deletion on normal hematopoiesis and leukemic microenvironment in B6.129S1-*Cd36*^*tm1Mfe*^/J Cd36-KO mice^25^. The role of APOC2 in normal hematopoiesis remains unknown.

APOC2 is a secreted protein, primarily expressed in the liver, but also produced by the intestine, macrophages, adipose tissue, whole blood, lungs, spleen and other tissues^4^. APOC2 binds to and activates LPL^10^, thus regulating lipid metabolism to provide energy transport and storage^6^. Due to its critical role, several *Apoc2*-KO and mutant mouse models have been developed to study lipid metabolism. Sakurai et al. generated *Apoc2*-mutant mice using zinc finger nucleases and observed that homozygous *Apoc2*-mutant mice exhibited mean TG levels comparable to those seen in *APOC2-*deficient patients. A marked increase in TG levels was shown in fractions corresponding to VLDL and chylomicrons compared with WT mice, while there was a smaller peak of cholesterol in HDL size fractions^11^. Gao et al. reported the birth of homozygous *Apoc2*-KO pups generated by CRISPR–Cas9 editing in a hamster model^10^. However, these pups displayed reduced body weight gain and markedly elevated TC and TG levels in *Apoc2*-KO compared with WT animals. In addition, half of them died by day 5, and all animals died within 9 days after birth^10^. Although neonatal death could be rescued by intravenous administration of normal hamster serum, about 25% of the treated hamsters did not survive to adulthood. Some phenotypes found in these homozygous *Apoc2*-KO pups were similar to those shown in *lpl*-deficient pups, which also died shortly after birth, as reported by Weinstock et al.^10,26^. Their findings suggested that the complete loss of LPL in rodents prevents TG removal from plasma, leading to the death of newborns^26^. *Lpl*-deficient pups exhibit poor ability to utilize the TG-rich milk from their mothers^27^. Due to the limitations of existing homozygous *Apoc2*-KO models for research applications, we hypothesized that inducing *Apoc2* KO after weaning could be a viable approach for studying the effects of *Apoc2* deletion in adult mice.

In this study, we developed a Cre-inducible *Apoc2-*KO mouse model, where global *Apoc2* deletion can be triggered by intraperitoneal TAM injection. This model provides the flexibility to control the timing of *Apoc2* deletion, enabling researchers to study its effects at different developmental stages. To enhance the generalizability of findings and ensure that the observed phenotypes are robust across developmental stages, mouse ages were matched before the start of experiments and both short-term and long-term experiments were designed to evaluate both immediate and prolonged effects of *Apoc2* deficiency. Multiple control groups and the strain background consistency were used to distinguish the *Apoc2*-KO phenotypes from the effects of TAM injection, Cre expression or genetic background. We monitored the body weight changes of eight mice from the same litter equally separated into *Apoc2-*KO group and WT group 3 weeks after the TAM injections, *Apoc2-*KO mice showed mildly higher body weight gain than their WT siblings injected with corn oil. The KO mice looked normal with no obvious impact on major organs. While KO mice exhibited high TG levels, they lived much longer than previously reported mouse models born with *Apoc2* deficiency. Four 18-month-old *Apoc2*-KO mice, including both males and females, that were injected with TAM at 3 months of age were still alive and healthy at the time of manuscript preparation.

Anemia and the disruption of the maintenance and differentiation of HSPCs were observed in both *apoc2*-KO zebrafish and *lpl*-KO zebrafish^28^. In *apoc2*-KO zebrafish, the total blood cell counts significantly decreased compared with the control group and Hb staining was weakened. In addition, the expressions of HSPC markers *runx1*/*cmyb* and of the blood lineage markers *beta-globin* (erythropoiesis) and *rag1* (lymphopoiesis) were significantly decreased in *apoc2*-KO zebrafish compared with WT, suggesting that HSPC expansion and differentiation were affected^28^. However, this nonmammalian animal model has limitations for modeling human blood and metabolic diseases, including differences in body temperature, the absence of brown adipose tissue limiting thermogenesis-related studies, the lack of human-equivalent tissues such as breast, prostate, lymph nodes, and bone marrow, anatomical differences and poor reproducibility across laboratories^29,30^. Therefore, the establishment of a mouse model and comprehensive investigation of the impact of *Apoc2* deficiency on normal hematopoiesis are required. Our studies show very low *Apoc2* expression in the BM of WT mice, which may explain the limited impact on normal hematopoiesis in *Apoc2-*KO mice. Our complete blood count analysis showed no significant differences in WBC, RBC, Hb or lymphocyte percentages between *Apoc2-*KO and WT mice, and Wright–Giemsa staining of mouse peripheral blood did not reveal different blood cell morphological changes or dysplasia after inducing *Apoc2* KO, indicating minimal effects of *Apoc2* KO on mouse hematological profiles. The BM cell proliferation assay, HSPC proliferation assay and colony-forming assay reinforced the undetectable impacts of *Apoc2* KO on the early hematopoietic hierarchy and normal hematopoiesis. Furthermore, *Apoc2* KO had a limited impact on splenocyte expansion in vitro in the presence of cytokines supporting the expansion of T cells. The expansion and repopulation ability of HSPCs were not significantly reduced by *Apoc2* KO.

Consistent with the limited impact on normal hematopoiesis, RNA-seq of BM cells showed few genes being differentially expressed upon *Apoc2* deletion. We identified five significantly upregulated genes and four significantly downregulated genes. Importantly, the heme metabolism pathway and associated *Gm16867* gene were significantly upregulated in the BM of *Apoc2-*KO mice. The E2F targets pathway was also upregulated. It is important to note that the sample size for the RNA-seq analysis was relatively small. However, these analyses were exploratory in nature and still support the conclusion that *Apoc2* deletion has a limited hematopoietic impact, probably due to its low expression in the BM. It will be important to further evaluate whether the proportions of various HSPC populations in *Apoc2-*KO BM differ from those in WT BM. Although *Apoc2-*KO mice can survive with elevated TG levels, their long-term survival should be monitored to identify any potential late-onset diseases or symptoms. Further histological analysis is needed to evaluate potential lipid accumulation or macrophage foam cell formation in the BM. In this study, *Apoc2*-KO mice exhibited lower HDL levels than WT mice. Due to the suppressive effect of HDL on HSPC proliferation, changes in HDL and gene expression in HSPCs following *Apoc2* KO induction will be further investigated. Future studies utilizing this model will focus on assessing the effects of *Apoc2* KO on aging-related phenotypes and leukemia progression, further establishing APOC2 as a potential therapeutic target in AML.

In conclusion, we successfully established a global *Apoc2*-KO mouse model using the Cre/*lox* system. Our results show that severe effects on hematopoiesis were not detectable in this study after inducing *Apoc2* KO. This Cre-inducible model provides the unique advantage of enabling *Apoc2* deletion after weaning, offering researchers a versatile tool to study the role of *Apoc2* in cancer and lipid metabolism in adult mice.

## Methods

### Generation of *Apoc2*^*fl/fl*^/*Gt(ROSA)26Sor*^*Cre/Cre*^ mice

All animal studies were approved by the Institution for Animal Care and Use Committee (IACUC) at the University of Southern California (USC). The study was in accordance with the ethical standards formulated in USC IACUC Animal Protocol Number 20581. Both male and female mice were included in the studies. C57BL/6J mice (RRID: IMSR_JAX:000664) were included in the WT control group and the CD45.2^+^ donors in the competitive repopulation assay. B6.SJL-*Ptprc*^*a*^ *Pepc*^*b*^/BoyJ mice (RRID:IMSR_JAX:002014) were used as CD45.1^+^ donors and crossed with C57BL/6J mice to get CD45.1^+^CD45.2^+^ heterozygous mice as recipients in the competitive repopulation assay. B6.129-*Gt(ROSA)26Sor*^*tm1(cre/ERT2)Tyj*^/J (R26-CreERT2) mouse strain (RRID:IMSR_JAX:008463) homozygous for *Cre* gene (*Gt(ROSA)26Sor*^*Cre/Cre*^) were ordered from The Jackson Laboratory. C57/BL6 mice homozygous for the floxed *Apoc2* gene (*Apoc2*^*fl/fl*^) were obtained from Dr. Alan Remaley’s lab at the National Heart, Lung, and Blood Institute at the National Institute of Health. *Gt(ROSA)26Sor*^*Cre/Cre*^ mice were then crossed with *Apoc2*^*fl/fl*^ mice to get the first-generation mice. Mice in the first generation were mated to generate the second generation, which then were mated to get the third generation of *Gt(ROSA)26Sor*^*Cre/Cre*^*Apoc2*^*fl/fl*^ mice. All *Apoc2*-KO and WT mice were bred on a consistent C57BL/6J and 129S4 mixed background to ensure genetic uniformity across groups. Control groups, including *Gt(ROSA)26Sor*^*wt/wt*^*Apoc2*^*fl/fl*^, *Gt(ROSA)26Sor*^*Cre/Cre*^*Apoc2*^*wt/wt*^ and C57BL/6J mice, were matched for strain background to account for potential strain-related variability. Mice were euthanized by CO_2_ asphyxiation followed by cervical dislocation.

### DNA extraction and genotyping

About 5–10 µL blood was collected from mouse tail vein into 20 µL 2% ethylenediaminetetraacetic acid (EDTA) (Invitrogen, cat. no. 15575020). The DNeasy Blood and Tissue kit (Qiagen, cat. no. 69504) was used to extract DNA following the manufacturer’s protocol. The DNA concentration was measured on NanoDrop One, and then DNA samples were stored at −20 °C.

The sequences of primers to confirm the presence or absence of *Cre* gene were obtained from The Jackson Laboratory. The primer sequences used to identify mutant *Cre* (*Cre*-MT) primer sequences were forward primer 5′-CGTGATCTGCAACTCCAGTC-3′ and reverse primer 5′-AGGCAAATTTTGGTGTACGG-3′. The primer sequences used to detect the WT gene (*Cre*-WT) were forward primer 5′-CTGGCTTCTGAGGACCG-3′ and reverse primer 5′-CCGAAAATCTGTGGGAAGTC-3′. The lengths of PCR-amplified products for *Cre*-MT and *Cre*-WT are 150 bp and 198 bp, respectively. In the R26-CreERT2 mouse strain, a cassette containing *Cre-ERT2* was inserted into the R26 locus. The mutant forward primer binds in the R26 allele just upstream of the cassette insertion site and the mutant reverse primer binds within *Cre* (Supplementary Fig. 1a). The mutant band can only amplify if the *Cre* construct is present. The WT forward primer binds in the *ROSA* allele upstream of where the insertion site would be, and the WT reverse primer binds within the section of the WT *ROSA* allele that is displaced by the mutation (Supplementary Fig. 1a). The WT primer set will amplify only if the *Cre* construct is absent. Two primer sets were designed to check the presence of the *lox*P site (*Apoc2-lox*) or WT (*Apoc2-*WT) in intron 4 of the *Apoc2* gene, respectively. The *lox* site in intron 4 was confirmed using the following primers: forward primer 5′-CTCTATCTCATGATTGCTGG-3′ and reverse primer 5′-CAGGATCCATAACTTCGTAT-3′. The expected PCR-amplified product length is 599 bp. The WT in intron 4 was confirmed by the following primers: forward primer 5′-TACCCGGATGTGGCAAGC-3′ and reverse primer 5′-AGAGGGTCTCATGTAGCC-3′. The expected PCR-amplified product length is 140 bp. The *Apoc2-lox* forward primer binds in the *Apoc2* gene just upstream of the *lox*P site in intron 4, and the *Apoc2-lox* reverse primer binds partially within the *lox*P sequence (Supplementary Fig. 1b). The *lox* band is only amplified if the *lox*P site in intron 4 is present. The *Apoc2-*WT forward primer binds within the WT *Apoc2* sequence in intron 4 where the *lox*P site would partially displace, and the reverse primer binds in the WT sequence after the insertion site (Supplementary Fig. 1b). The *Apoc2*-WT band is only amplified if the WT *Apoc2* sequence is present.

Gradient PCR was done to optimize the annealing temperature for primers and 57.7 °C was found to be the most suitable temperature. Each 25-μl reaction mixture consisted of >10 ng DNA, DNase/RNase-free water (ThermoFisher), forward and reverse primers, and Taq 2X Master Mix (NEB). PCR products were resolved on a 1% agarose gel (IBI Scientific) stained with ethidium bromide (ThermoFisher) and electrophoresed at 100 V alongside a 1 kb Plus DNA ladder (NEB). Genotypes were confirmed by visualizing bands using either the iBright FL1000 or ChemiDoc Touch Gel Imager (ThermoFisher/Bio-Rad). WT, heterozygous and homozygous genotypes of the *Cre* gene (Supplementary Table 3) and *lox* site (Supplementary Table 4) can be confirmed by the presence and the number of bands.

### TAM-induced *Apoc2* KO in vivo

TAM (20 mg/mL in corn oil, Sigma-Aldrich, cat. nos. T5648 and C8267) was prepared by stirring overnight at 37 °C, protected from light, and stored at 4 °C. Mice (1–3 months old, unless otherwise specified) received intraperitoneal TAM at 75 mg/kg daily for 5 consecutive days. The experimental group (*Gt(ROSA)26Sor*^*Cre/Cre*^*Apoc2*^*fl/fl*^) and control WT group were injected with either TAM or corn oil as shown in Supplementary Table 1. In the WT control group, *Gt(ROSA)26Sor*^*wt/wt*^*Apoc2*^*fl/fl*^ and C57BL/6J mice were injected with TAM to account for TAM’s side effects and ensure the observed phenotypes in *Apoc2*-KO mice result from gene KO rather than the TAM; *Gt(ROSA)26Sor*^*Cre/Cre*^*Apoc2*^*wt/wt*^ mice were injected with TAM to control for the potential effects of TAM-induced Cre expression; *Gt(ROSA)26Sor*^*Cre/Cre*^*Apoc2*^*fl/fl*^ mice were injected with corn oil (vehicle) to confirm that the genetic background of *Apoc2*-KO mice does not independently contribute to observed phenotypes. *Apoc2*-KO and WT mice were age-matched at the start of experiments with slightly varying ages across experiments. Mice were euthanized by CO_2_ asphyxiation followed by cervical dislocation at different time points, at least 11 days after the last TAM injection. Both short-term and long-term experiments were designed to evaluate immediate and prolonged effects of Apoc2 deficiency. Tissues (blood, liver, spleen and BM) were collected.

### Quantitative real-time PCR analysis

RNA extractions from liver, spleen, BM and blood tissues were performed following TRIzol (Invitrogen, cat. no. 15596018) and chloroform extraction protocol. Equal amounts of RNA were then used for SuperScript IV reverse transcription to synthesize cDNA, following the manufacturer’s protocol (Invitrogen, cat. no. 18091050). The qPCR was performed using Taqman gene expression assay to quantify the *Apoc2* gene (Taqman, cat. no. Mm00437571_m1) and reference mouse housekeeping gene *B2m* (Taqman, cat. no. Mm00437762_m1). Each 8-µL PCR reaction mixture contained 2 µL cDNA per well in a 384-well plate and was run in triplicate using the ABI QuantStudio 12K Flex Real-Time PCR System (Applied Biosystems), following the manufacturer’s instructions. The relative transcripts levels of *Apoc2* mRNA were normalized to *B2m* and calculated using the 2^−∆∆CT^ method.

### Hematological laboratory analysis

About 50 µL mouse heart blood was collected immediately after euthanasia by CO_2_ asphyxiation followed by cervical dislocation in a MiniCollect K3 EDTA tube (Greiner Bio-One, cat. no. 450475), and then the complete blood count test was performed in the Element HT5 Veterinary Hematology Analyzer. The blood analysis included WBC count, RBC count, Hb, HCT, MCV, MCH, MCHC, platelets and the absolute value and percentage of neutrophils, lymphocytes, monocytes, eosinophils and basophils. To examine the differentiation of morphological cell types of peripheral blood, fresh tail blood was collected from four male *Apoc2*-KO mice and four male WT mice (Supplementary Table 1, experiment 13). Peripheral blood smears were done by Quick Stain kit (VWR, cat. no. 10143) to examine the differentiation of morphological cell types. Blood slides were immersed and processed for 30–60 s in Wright–Giemsa stain solution in a staining dish. Then, blood slides were immersed into deionized water for 2 min. After rinsing with deionized water and allowing it to dry, slides were examined under 20× Zeiss Scope A1 microscope.

### TG and TC colorimetric assays

Mouse whole blood was clotted under room temperature without disturbing for at least 30 min, and then was centrifuged at 1,000*g*, 4 °C for 20 min. The serum was collected from the supernatant instantly after centrifuging and stored at −80 °C. Thawed serum samples were kept on ice and used within 2 h. Serum samples were diluted with Dulbecco’s phosphate-buffered saline (ThermoFisher, cat. no. 14190250) to keep the optical density (OD) value within the range of the standard curve. The concentration of TG and TC were measured by TG colorimetric assay kit (ThermoFisher, cat. no. EEA028) and TC colorimetric assay kit (ThermoFisher, cat. no. EEA026) respectively. OD values were measured under BioTek Synergy H1 Hybrid Multi-Mode Microplate Reader (Agilent), and then the concentrations were calculated using the formula provided by the kit. Serum samples were extracted at random time points after injection, including 11 days, 1 month or 5 months. Also, serum was also collected on days 1, 7, 14 and 22 after injection to monitor changes of TG and TC levels in four *Apoc2*-KO mice and five WT mice.

### Plasma lipoprotein profile analysis

Pooled mouse plasma samples (*n* = 2 M + 2 F each group, experiment 11) were filtered through 0.45-μm Amicon-Ultra spin filters, and 250 μL (Female samples) or 140 μL (male samples) were loaded into Superose 6 Increase columns (GE Scientific) on the AKTA pure FPLC system (GE Scientific). Each 0.5-mL fraction was tested for phospholipids using colorimetric assays (Fujifilm Wako). All WT mice were C57BL/6J CD45.2^+^ background. Two males were injected with TAM at 2 months of age and euthanized 5 months post-injection. Two CD45.2^+^ females were injected with TAM at 1 month of age and euthanized 7 months post-injection. While two *Apoc2*-KO males were injected at 1 month of age and had a 7-month waiting period, two females were injected at 2 months of age and waited 5 months before plasma collection.

### Cell proliferation assay

Mice (*n* = 5 in *Apoc2-*KO group, *n* = 4 in WT group) from experiments 1, 2 and 5 were included for the assessment of in vitro expansion of splenocytes (T cells) and BM hematopoietic stem cells from *Apoc2-*KO and WT mice over 10 days. We started with about 2 × 10^6^ cells per well and 2 technical replicates per mouse. The BM cells were cultured in 20% fetal bovine serum-RPMI (R20) (ThermoFisher, cat. no. 11875093) supplemented with SCF (Peprotech, cat. no. AF-250-03) with a concentration of 10 ng/mL and TPO (Peprotech, cat. no. 315-14) with a concentration of 100 ng/mL. Cell proliferation assays were also conducted on the whole BM cells cultured with 10 ng/mL SCF, 100 ng/mL TPO, 10 ng/mL IL-3 (Stemcell, cat. no. 78042) and 10 ng/mL IL-6 (Stemcell, cat. no. 78052) for a total of 6 days. In the four-cytokine proliferation assay, two male *Apoc2*-KO and two male WT mice were euthanized by CO_2_ asphyxiation followed by cervical dislocation about 6 months post-injection (experiment 9). The mouse primary spleen cells were cultured in R20 supplemented with 2.5 ng/mL IL-2 (RD System, cat. no. 402-ML) and 1.25 μg/mL PHA (ThermoFisher, cat. no. 00-4977-03). The trypan blue cell counting assay (ThermoFisher, cat. no. 15250061) was performed two times per well on days 1, 2, 4, 6, 8 and 10 to obtain the total number of viable cells per milliter of cell suspension medium. On days 5 and 10, 90 μL cell suspension were mixed with 10 μL CCK8 (abcam, cat. no. ab228554) reagent with four technical replicates per mouse for CCK8 assay as another method to monitor cell proliferation, and 90 μL R20 mixed with 10 μL CCK8 reagent with three technical replicates served as control background. After incubating at 37 °C overnight, OD values were measured on BioTek Synergy H1 Hybrid Multi-Mode Microplate Reader (Agilent) and the relative in vitro cell viability values were calculated. The CCK8 assay was repeated on day 6, with incubation at 37 °C for at least 4 h, when BM cells had proliferated in the presence of four cytokines. A 9-day CellTrace assay was performed to monitor proliferation of BM cells and splenocytes by dye dilution, with cells plated on the same day as those used for the cell counting assay. About 2 μM CellTrace CFSE dye was used to stain 1 million cells. We started with about 2 × 10^6^ cells per well, including two stained replicates and one unstained control well per mouse. CellTrace CFSE dye dilution was tracked by flow cytometry on days 1, 3, 6 and 9. To study the effects of *Apoc2* on early hematopoietic hierarchy, HSPCs were isolated from BM cells and their proliferation ability was tested (*n* = 1 M + 1 F per group; Supplementary Table 1, experiment 12). Two WT mice were C57BL/6J CD45.2^+^ mice injected with TAM. One *Apoc2*-KO male and WT male received additional 5-fluorouracil injection 4 days before euthanasia. HSPCs were cultured in R20 supplemented with 10 ng/mL SCF, 50 ng/mL TPO, 10 ng/mL IL-3 and 10 ng/mL IL-6 and tracked for up to 6 days by trypan blue cell counting.

### Colony-forming cell assay

Approximately 50,000 BM cells were added to ~1 mL of mouse methylcellulose complete medium (BD, cat. no. HSC007), which is designed to support the in vitro growth of HSPCs. Cells were plated on a 6-well suspension culture plate with 35 mm diameter. Two replicates per mouse were included, and the colony-forming units (CFU) were counted twice per well under a microscope on day 7.

### Competitive repopulation assay

The whole BM cells were collected from the femurs of CD45.1^+^ WT mice, CD45.2^+^ WT mice, and CD45.2^+^ *Apoc2-*KO mice. Donor mice from experiment 3 were males injected with TAM for 5 consecutive days at the same time and euthanized by CO_2_ asphyxiation followed by cervical dislocation after 1 month. Because long-term engraftment and hematopoietic reconstitution primarily result from undifferentiated, multipotent hematopoietic stem cells rather than differentiated cells with limited repopulation capacity, specific HSPC isolation or additional hematopoietic stem cell marker staining was not performed. CD45.1^+^ WT whole BM cells were mixed with CD45.2^+^*Apoc2-*KO whole BM cells at 1:1 ratio to make the mixture of donor cells. Approximately 1.4 × 10^7^ mixed donor cells were transplanted via intravenous injection into 5–6-month-old CD45.1^+^CD45.2^+^ heterozygous recipient mice including two male mice and one female mouse. In addition, CD45.1^+^ WT BM cells were also mixed with CD45.2^+^ WT BM cells at a 1:1 ratio (7 × 10^6^ BM cells per donor) to be transplanted into 5–6-month-old CD45.1^+^CD45.2^+^ heterozygous recipient mice including one male mouse and one female mouse. This experiment served as a control to rule out differences in HSPC reconstitution ability between CD45.1^+^ and CD45.2^+^ backgrounds. The competitive repopulation assay was repeated with the same number of donor cells from two *Apoc2-*KO mice and two *Gt(ROSA)26Sor*^*wt/wt*^*Apoc2*^*fl/fl*^ mice in WT group 1 month after TAM injection in experiment 4 (*n* = 1 M + 1 F per group) and the same waiting time. In the KO group, *Apoc2-*KO BM cells were mixed with CD45.1^+^ WT BM cells at a 1:1 ratio to be transplanted into 2-month-old CD45.1^+^CD45.2^+^ irradiated mice (*n* = 2 M + 1 F). In the WT group, BM cells were collected from *Apoc2*^*fl/fl*^ mice (no Cre) injected with TAM and then mixed with CD45.1^+^ WT BM cells to be transplanted into CD45.1^+^CD45.2^+^ irradiated mice (*n* = 2 M + 1 F). All recipient mice received two separate single irradiation doses of 450 cGy over 24 h before BM transplant. All recipient mice were euthanized on day 34 after transplantation. The reconstitution for CD45.1^+^WT versus *Apoc2-*KO HSPCs and CD45.1^+^ WT versus CD45.2^+^ WT HSPCs in BM, spleen and blood was assessed by flow cytometry.

### Flow cytometry analysis

For the CellTrace assay, about 100 µL cells were collected from every well to run the flow cytometry. The reduced fluorescent intensities represented the CellTrace CFSE dye dilution tracked by flow cytometry. Flow cytometry analysis was also used to assess the BM engraftment in a competitive repopulation assay. BM, spleen and blood cells were collected from CD45.1^+^CD45.2^+^ heterozygous recipient mice, and 100 µL phosphate-buffered saline was used to prepare the dye solution with 1.25 µL CD45.1 monoclonal antibody (A20) (Invitrogen cat. no. 17-0453-81), and 0.5 µL CD45.2 monoclonal antibody (104) (Invitrogen cat. no. 11-0454-81) for each sample. Cells were incubated on ice protected from light for 30 min. Cells were then washed three times with phosphate-buffered saline and analyzed by flow cytometry to measure APC-conjugated CD45.1 and FITC-conjugated CD45.2.

### RNA-seq analysis

A total of eight BM RNA samples from two female mice (experiment 5) and two male mice (experiment 6) from each of the *Apoc2-*KO group and WT group were sent to Azenta for RNA-seq analysis. The integrity of RNA was measured by Agilent high-sensitivity RNA ScreenTape (Agilent Technologies, cat. no. 5067-5579) on the 4200 TapeStation System (Agilent Technologies, cat. no. G2991BA). RNA samples were stored at −80 °C before they were sent to Azenta for RNA-seq. Basic bioinformatic analyses were provided by Azenta which include raw RNA-seq gene counts, normalized RNA-seq gene counts and next-generation sequencing differential gene expression analysis report. GSEA^31,32^ was run to analyze the normalized RNA-seq gene counts to generate the gene enrichment plots and calculate NES. The parameters included Hallmark database (mh.all.v2023.2.Mm.symbols.gmt) and chip platform (Mouse_gene_Symbol_Remapping_MSigDB.v2023.2.Mm.chip).

### Statistical analysis

Each sample had three technical replicates in the qPCR analysis, and the mRNA levels in *Apoc2*-KO mice were normalized to those of the WT group in each experiment. The cell proliferation assay and CellTrace assay were repeated three times. Cell numbers or the MFI per day were normalized to the first day in each experiment and then were compared daily by multiple unpaired *t*-test (false discovery rate (FDR) approach, two-stage step-up method of Benjamini, Krieger and Yekutieli) between WT and *Apoc2-*KO groups. These two assays were done with two replicates per mouse, and the average value of these two replicates was calculated as the final value for that mouse and compared with mice in another group. In the CCK8 assay, cells were collected from the cell proliferation plate, and the assay was performed with four technical replicates per mouse. The cell viability of *Apoc2-*KO cells was normalized to that of WT group, and the significant difference was analyzed by unpaired *t*-test. In the competitive repopulation assay, the CD45.2^+^ WT/CD45.1^+^ WT or *Apoc2-*KO/CD45.1^+^ WT ratio per recipient was calculated and compared by unpaired *t*-test. This experiment was conducted twice. The Shapiro–Wilk test was run to evaluate the normality of data. An unpaired *t*-test was used to analyze normally distributed data; otherwise the Mann–Whitney test was used. The analysis results with a *P* value of less than 0.05 were considered significant. Data are presented as mean ± standard deviation (s.d.). GraphPad Prism 10 and R were used for statistical analysis and generating figures. For the RNA-seq data, the Wald test was used to generate *P* values and log_2_ fold changes. Genes with an adjusted *P* value <0.05 and absolute log_2_ fold change >1 were identified as differentially expressed genes. An FDR below 25% was considered significant.

### Reporting summary

Further information on research design is available in the Nature Portfolio Reporting Summary linked to this article.

## Online content

Any methods, additional references, Nature Portfolio reporting summaries, source data, extended data, supplementary information, acknowledgements, peer review information; details of author contributions and competing interests; and statements of data and code availability are available at 10.1038/s41684-026-01748-z.

## Supplementary information

Supplementary Information Supplementary Figs. 1–8 and Tables 1–4.

Reporting Summary

Supplementary Data 1 Statistical source data for Supplementary Figs. 2, 3 and 7.

## Source data

Source Data Fig. 1 Statistical source data.

Source Data Fig. 2 Statistical source data.

Source Data Fig. 3 Statistical source data.

Source Data Fig. 4 Statistical source data.

Source Data Fig. 5 Statistical source data.

Source Data Fig. 6 Statistical source data.

Source Data Fig. 7 Statistical source data.

Source Data Fig. 8 Statistical source data.

## Data Availability

All the data relevant to the study results are included in Figs. 1–8 or in Supplementary Information. Source data are provided with this paper.
