# Supplementary material for: Establishment and functional analysis of an inducible Apoc2-knockout mouse model to investigate the role of Apoc2 in normal hematopoiesis
Source: Lab Anim (NY). 2026 Jul 10;55(9):354–66. doi: 10.1038/s41684-026-01748-z (PMC13518242; doi:10.1038/s41684-026-01748-z)
Supplement: Supplementary file 1 [file 41684_2026_1748_MOESM1_ESM.pdf]

lab animal

Supplementary information

https://doi.org/10.1038/s41684-026-01748-z

Establishment and functional analysis of an inducible *Apoc2*-knockout mouse model to investigate the role of *Apoc2* in normal hematopoiesis

In the format provided by the authors and unedited

Establishment and Functional Analysis of an Inducible *Apoc2* Knockout Mouse Model to Investigate Apoc2's Role in Normal Hematopoiesis

Authors: Xiaochen Zhang[1], Ritu Chandwani[1], Mateusz Pospiech[1], Yiting Meng[1], Kanaka Dhuri[1], Atham Ali[1], Bhakti Thakker[1], Shephali Milind Kadam[1], Wenda Zhu[1], Yiyu Xiao[1], Denis Sviridov[2], Anna Wolska[2], Alan T. Remaley[2], Houda Alachkar[1,3,*]

Authors Affiliations:

[1] Department of Clinical Pharmacy, Alfred E. Mann School of Pharmacy and Pharmaceutical Sciences, University of Southern California, Los Angeles, CA 90089, USA

[2] Lipoprotein Metabolism Laboratory, Translational Vascular Medicine Branch, National Heart, Lung, and Blood Institute, National Institutes of Health, Bethesda, MD 20892, USA

[3] USC Norris Comprehensive Cancer Center, University of Southern California, Los Angeles, CA 90033, USA

***Corresponding author:**

Houda Alachkar, alachkar@usc.edu

USC Alfred E. Mann School of Pharmacy and Pharmaceutical Sciences

University of Southern California

1985 Zonal Avenue, PSC 608

Los Angeles, CA 90089

Supplementary information

Additional file 1

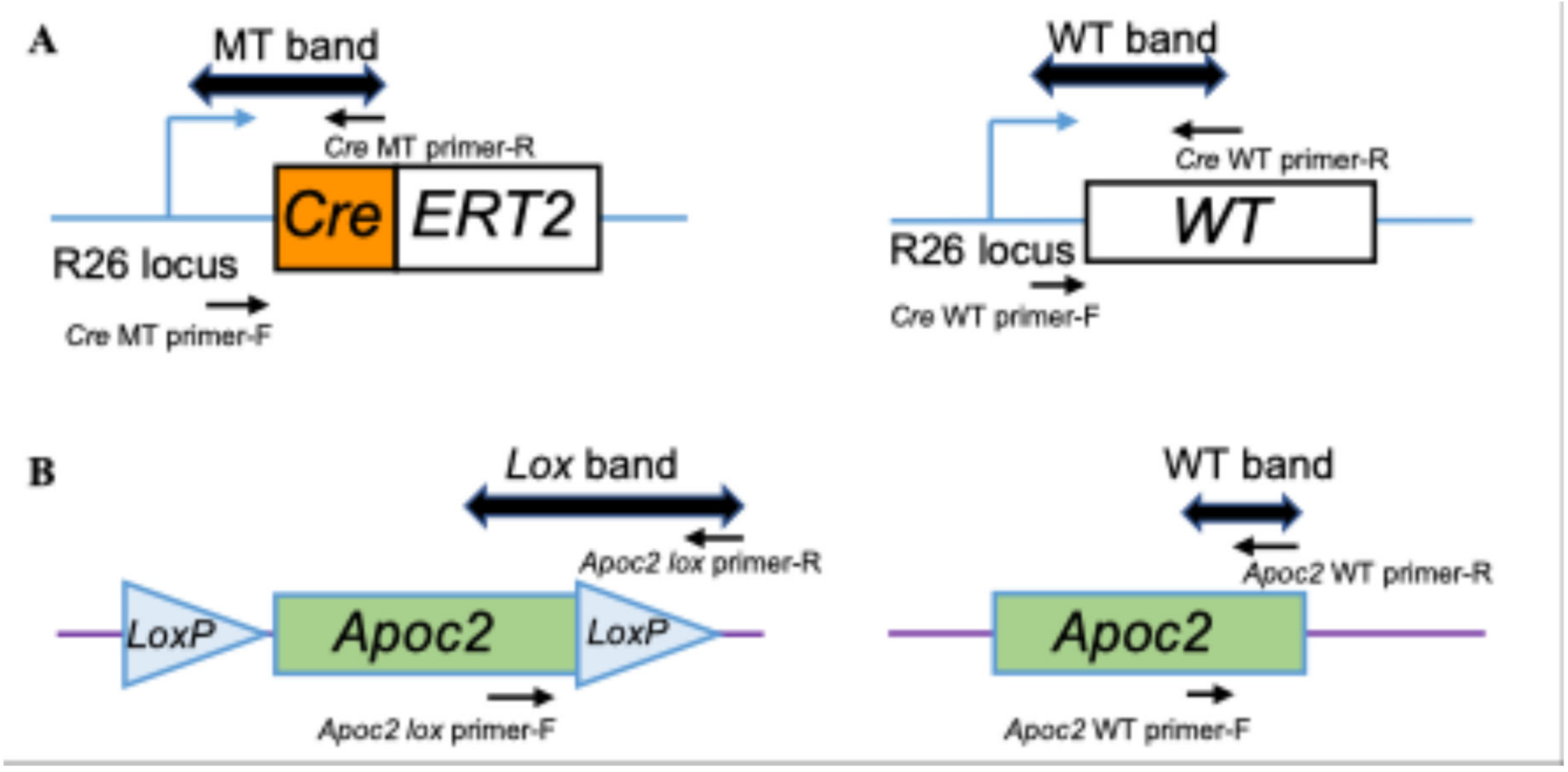

Figure S1. Schematic depiction of PCR reactions

(A) PCR reactions with *Cre*-MT primers or *Cre*-WT primers. (B) PCR reactions with *Apoc2-lox* primers or *Apoc2*-WT primers

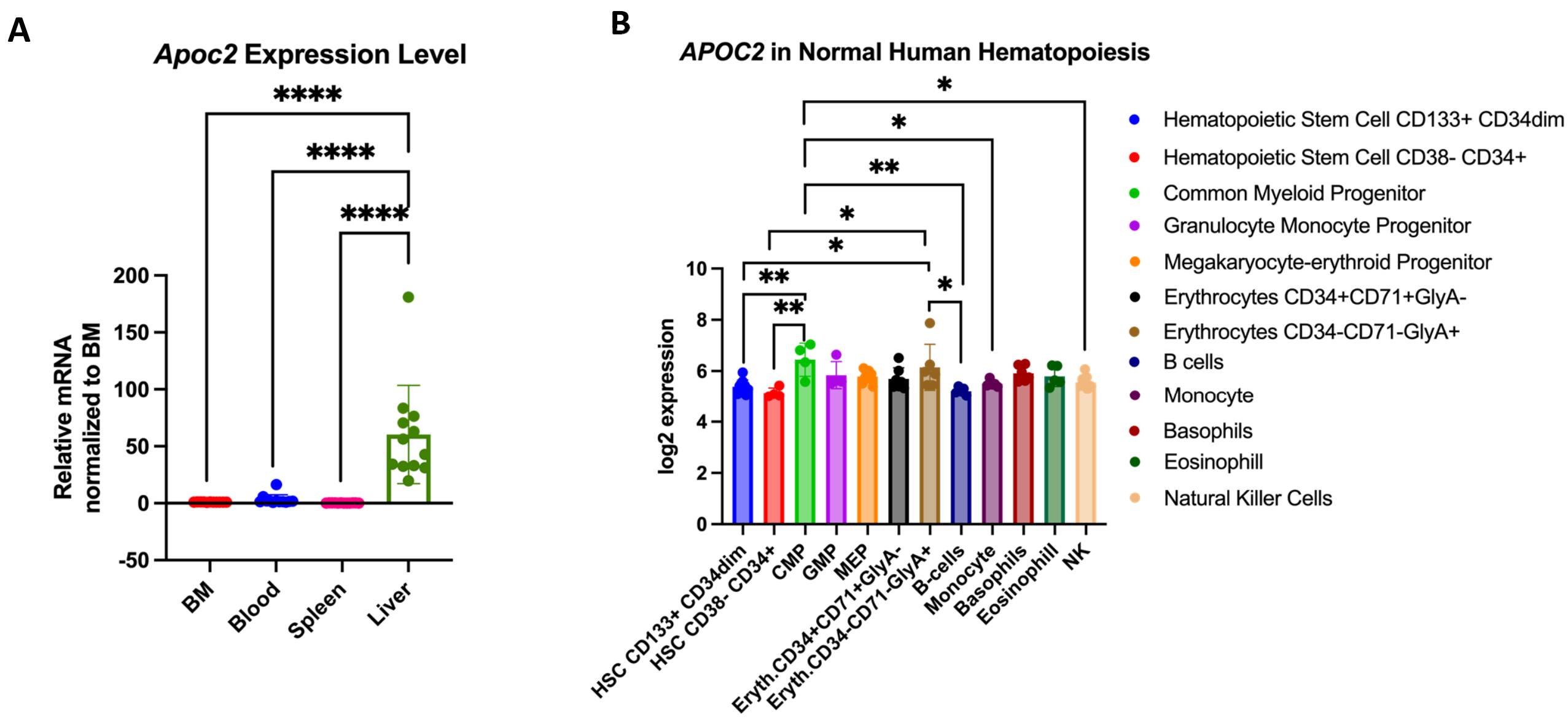

Figure S2. *Apoc2* is predominantly expressed in the liver of WT mice and is expressed across normal human hematopoietic cell types.

(A) The *Apoc2* mRNA levels in BM (n = 11), blood, spleen, liver (n = 12 each tissue) from WT mice were normalized to BM and compared. The bar graph shows the mean with standard deviation of *Apoc2* mRNA level per group. Each single dot represents the count for every single mouse. The difference was analyzed by one-way ANOVA. (B) The *APOC2* expression data was downloaded from the BloodSpot normal human hematopoiesis (DMAP) database. The log2 transformed expression level for *APOC2* in different cell populations is analyzed by One-way ANOVA (Abbreviation: ****, P < 0.0001; *, P < 0.05; ns, not significant).

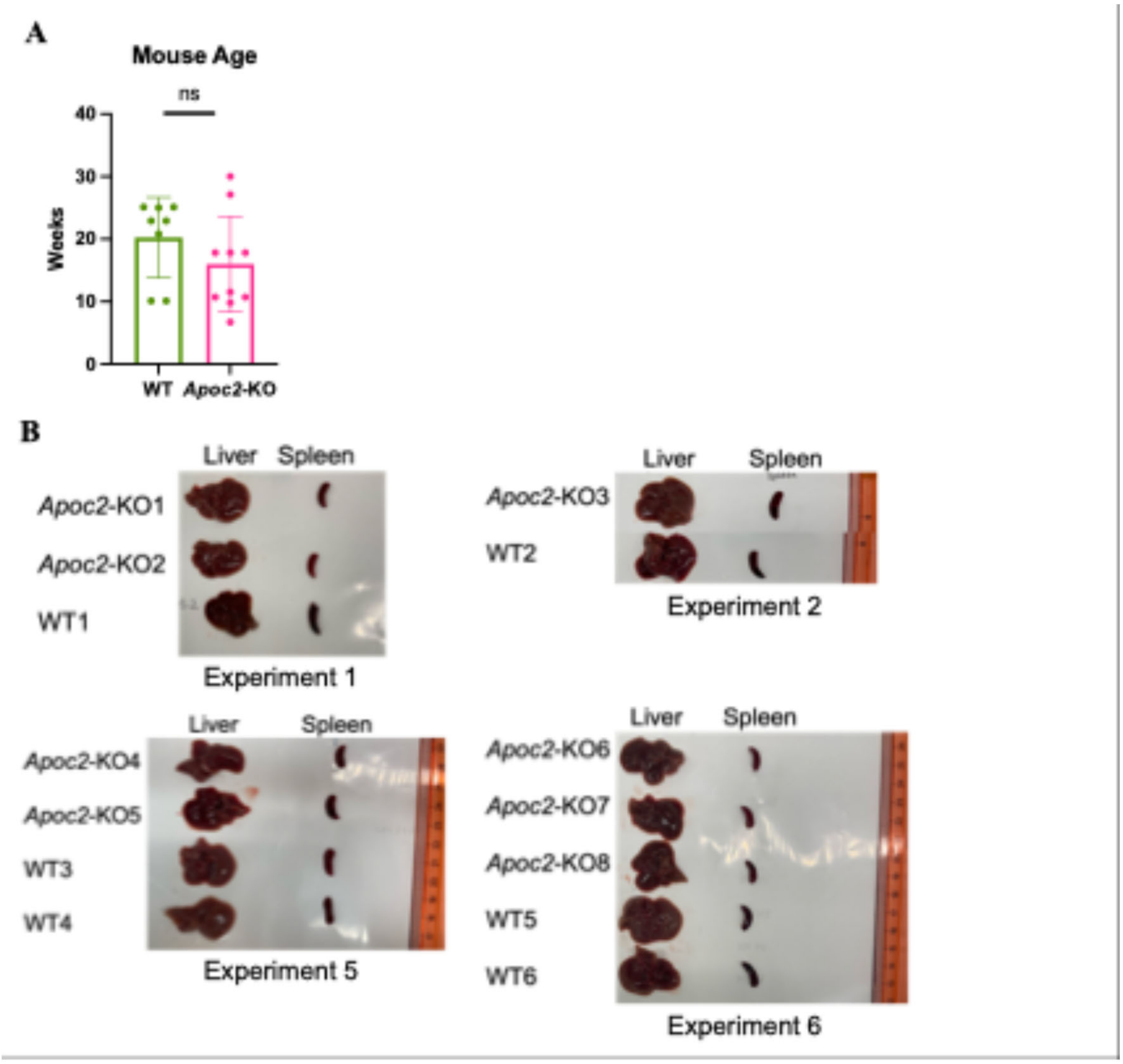

Figure S3. Liver and spleen tissues exhibited similar color and morphology in *Apoc2*-KO and the WT mice

(A) The mean age of *Apoc2*-KO mice (n = 10) and the WT mice (n = 8) that were included in the in vitro assays (*Apoc2*-KO vs WT = 15.99 weeks vs 20.25 weeks, P = 0.303; Abbreviation: ns, not significant). (B) In Experiment 1, 2, 5, and 6, liver and spleen tissues were collected from *Apoc2*-KO and WT mice after dissection. The color and shape were compared between the two groups.

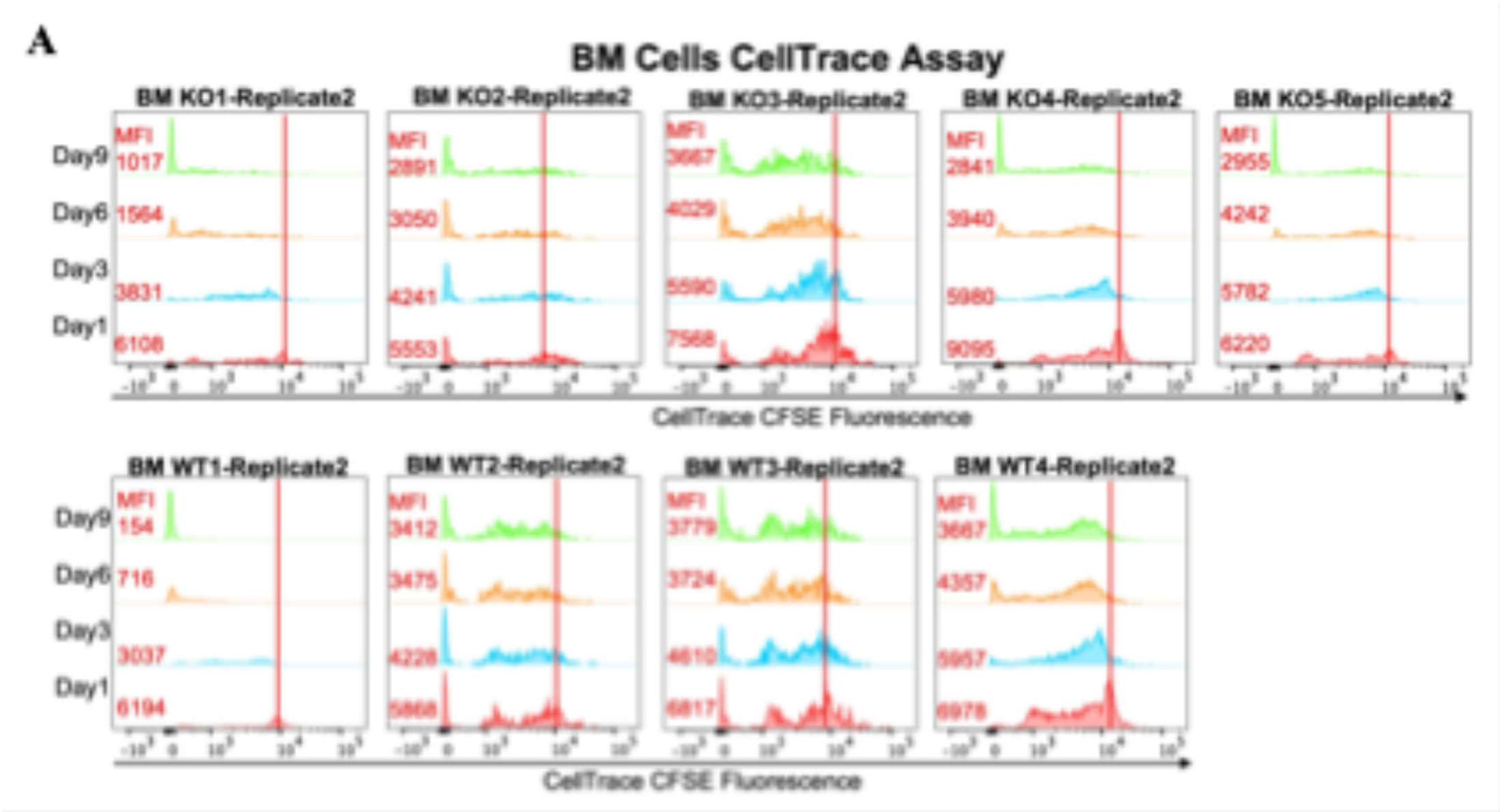

Figure S4. BM cells CellTrace assay did not show significant difference between WT group and *Apoc2*-KO group

(A) The CFSE fluorescence histograms of the second BM cells CellTrace replicate of every mouse. The mean fluorescence intensity value on day 1, day 3, day 6, day 9 was shown on every figure.

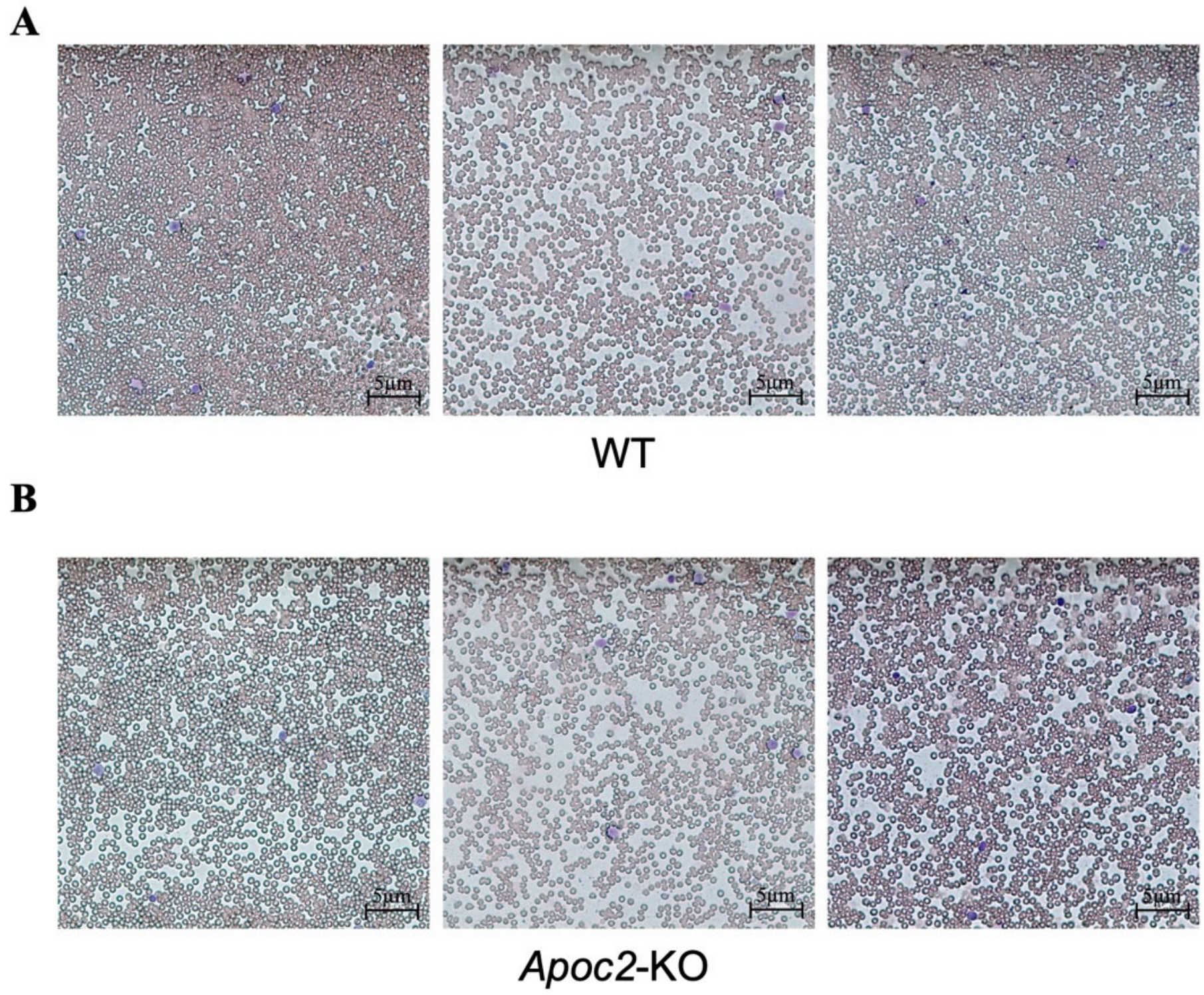

Figure S5. Wright-Giemsa staining of mouse tail blood showed no difference in cell morphologies between WT mice and *Apoc2*-KO mice

(A-B) Fresh tail blood smear of three WT males (A) and three *Apoc2*-KO males (B) were examined by Wright-Giemsa staining under 20x Zeiss Scope A1 microscope. One representative picture was selected for each mouse. Scale bar: 5μm.

A

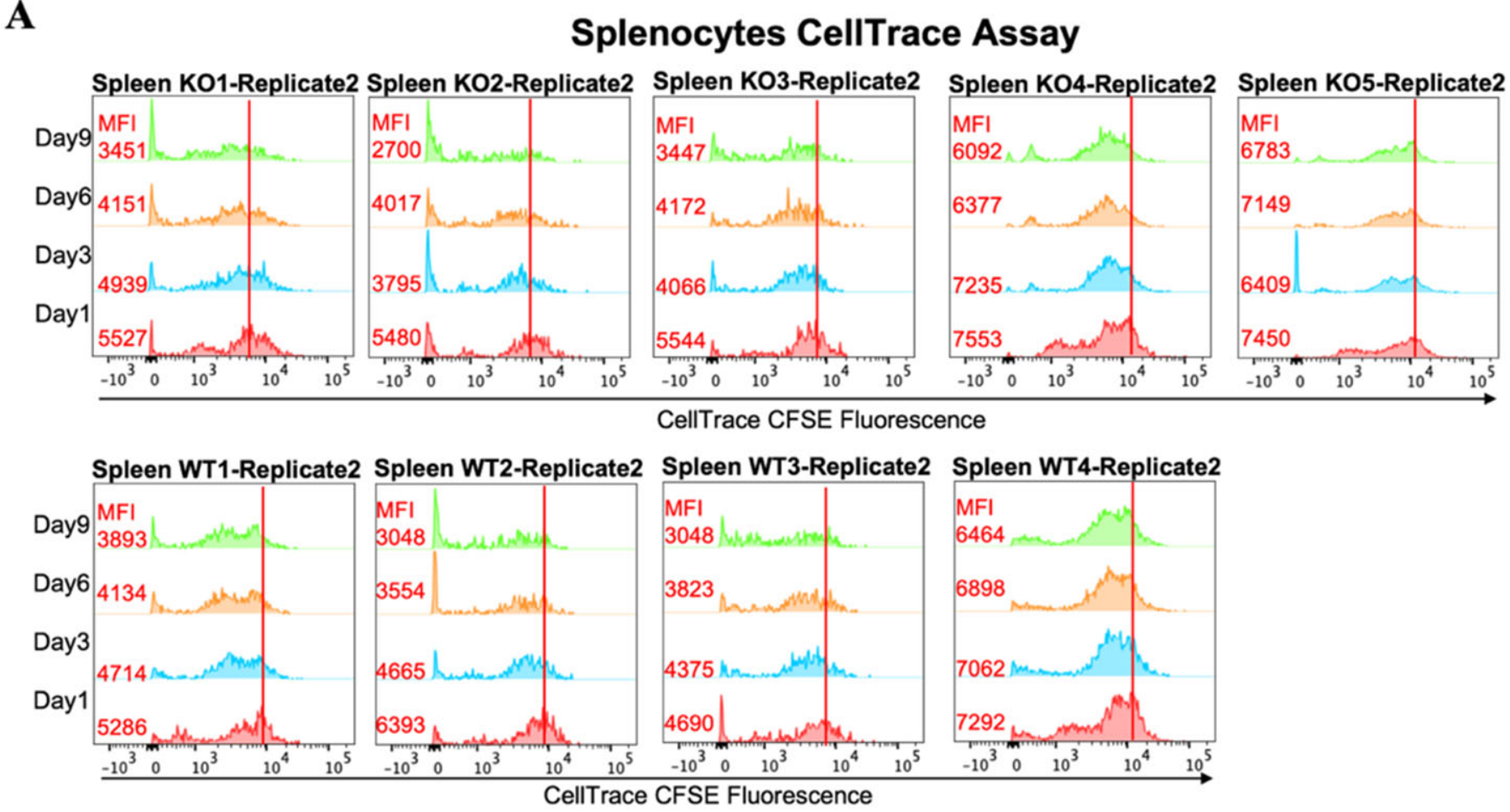

Figure S6. Splenocytes CellTrace assay did not show significant difference between the WT and the *Apoc2*-KO group

(A) The CFSE fluorescence histograms of the second splenocytes CellTrace replicate of every mouse. The mean fluorescence intensity value on day 1, day 3, day 6, day 9 was shown on every figure.

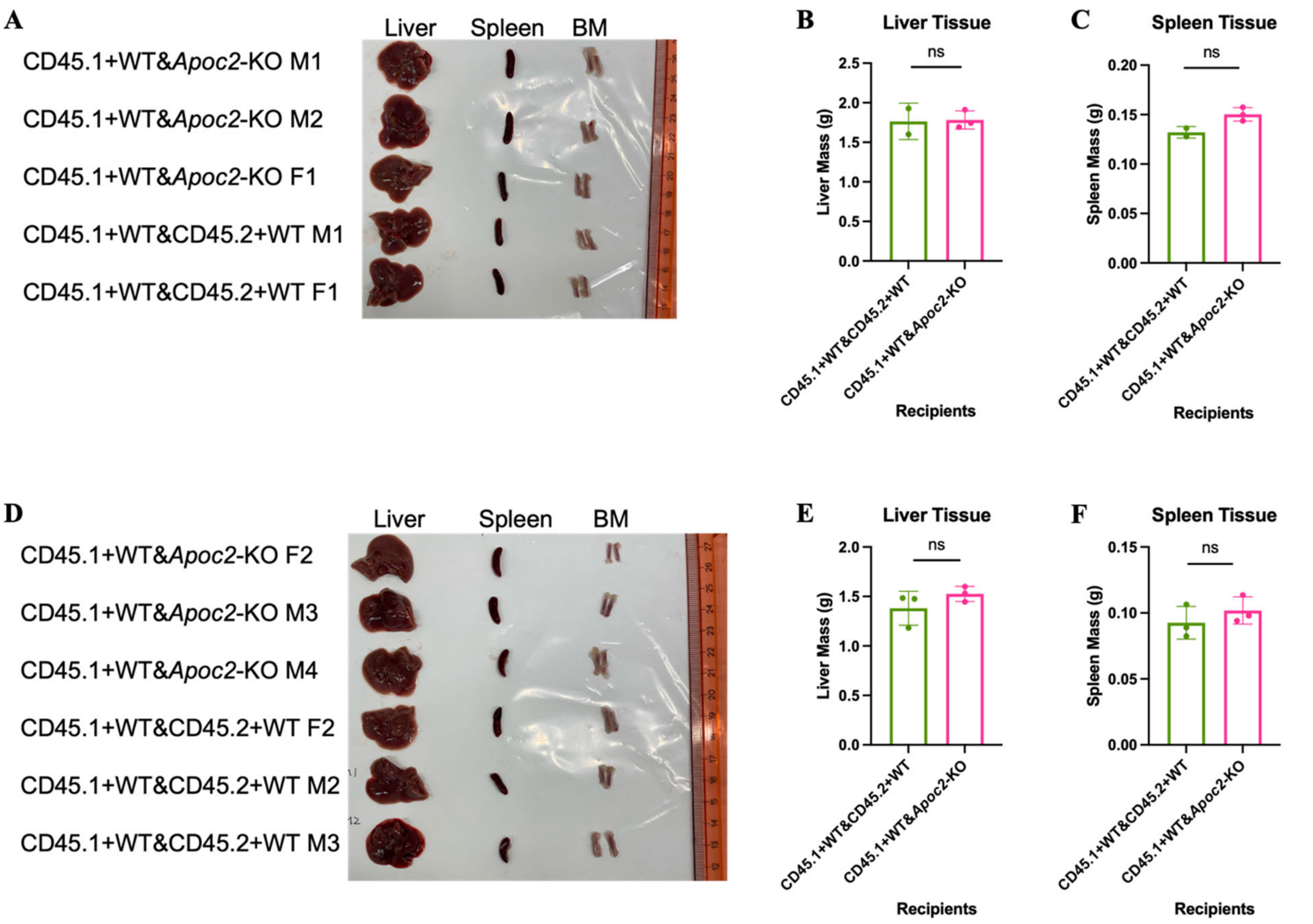

Figure S7. Liver and spleen tissues showed comparable morphology and weights between mice engrafted with WT and *Apoc2*-KO cells.

(A) Liver tissue, spleen tissue and BM of recipient mice were collected after euthanasia (n = 3 *Apoc2*-KO&CD45.1$^+$WT recipients, 2 CD45.2$^+$WT &CD45.1$^+$WT recipients). (B-C) Similar liver mass and spleen mass was observed between *Apoc2*-KO&CD45.1$^+$WT recipients and CD45.2$^+$WT &CD45.1$^+$WT recipients (*Apoc2*-KO &CD45.1$^+$WT vs CD45.2$^+$WT &CD45.1$^+$WT = liver: 1.78 g vs 1.76 g, P = 0.91; spleen: 0.15 g vs 0.13 g, P = 0.055). (D) Liver tissue, spleen tissue and BM of recipient mice in the repeated experiment were collected after euthanasia (n = 3 per group). (E-F) Similar liver mass and spleen mass was observed between *Apoc2*-KO &CD45.1$^+$WT recipients and CD45.2$^+$WT&CD45.1$^+$WT recipients (*Apoc2*-KO &CD45.1$^+$WT vs CD45.2$^+$WT

&CD45.1^{+}WT = liver: 1.53 g vs 1.38 g, P = 0.26; spleen: 0.10 g vs 0.093 g, P = 0.38; Abbreviation: ns, not significant).

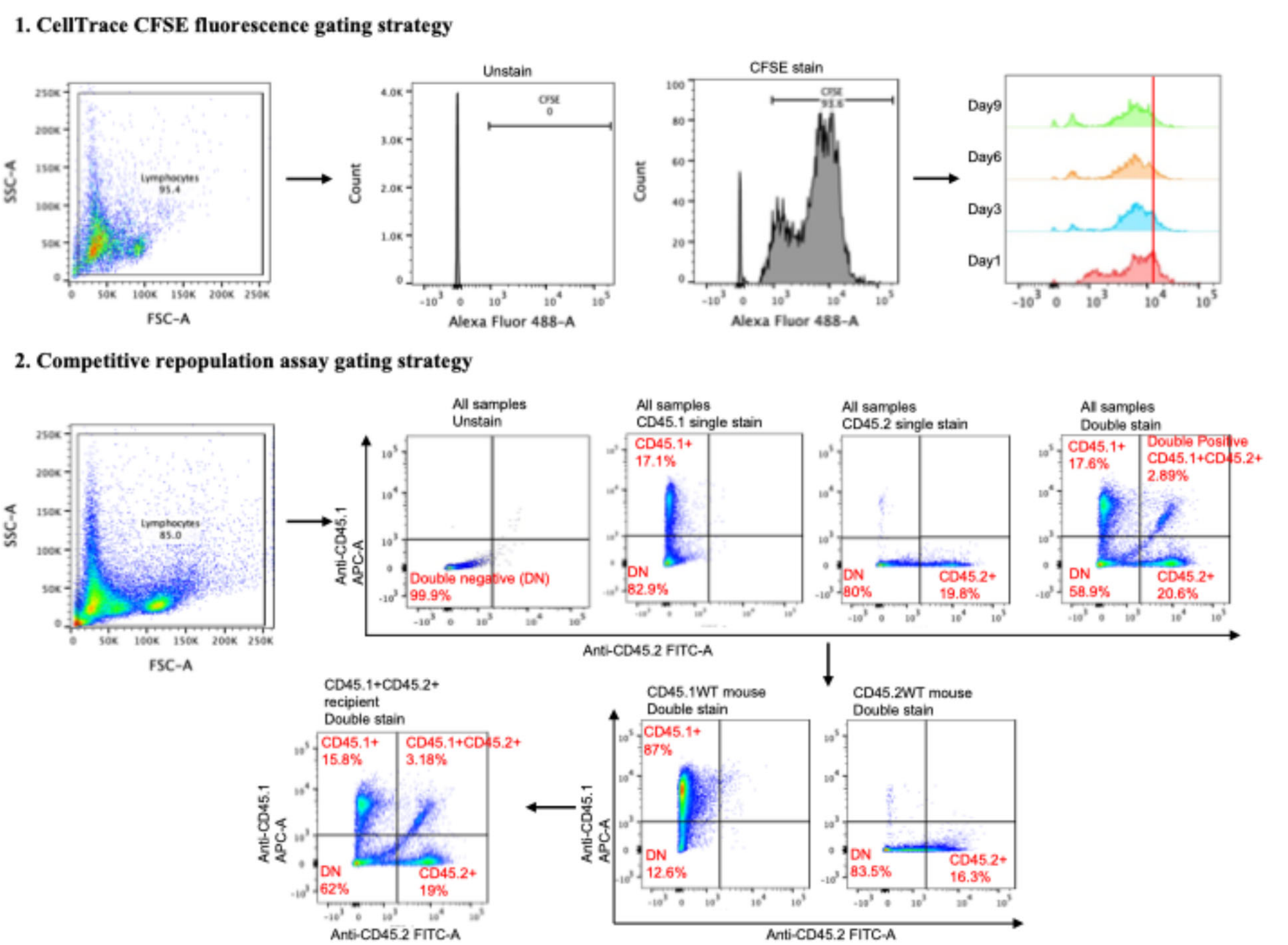

Figure S8. Flow cytometry gating strategies

CFSE staining was used to track the proliferation of cells. Each mouse had one unstained flow sample and two replicates with CFSE staining. Unstained sample was used to gate the CFSE fluorescence. The histograms of four days were put in the same figure as offset style to compare the shift of the peak. When run the competitive population assay, the same volume of each cell sample suspension was collected into one tube, mixed and split to four replicates, unstained tube, CD45.1 dye single-stained tube, CD45.2 dye single-stained tube, CD45.1 and CD45.2 double-

stained tube, respectively. The APC-FITC results were used to gate the double negative (DN), CD45.1 single positive (CD45.1^{+}), CD45.2 single positive (CD45.2^{+}), and double positive (CD45.1^{+}CD45.2^{+}) range. CD45.1^{+} WT mouse and CD45.2^{+} WT mouse were included as staining control and gating reference.

Table S1. Tamoxifen induced *Apoc2* knockout experimental plan, all the mice were ~ 1-3 months old at the time of TAM injection unless otherwise stated in the table.

Experiment	Group	Mice Gender	Mice Number and Genotype	Injection	Experiment Process
Experiment 1	*Apoc2*-KO	Female (F)	1*Gt(ROSA)26Sor*$^{Cre/Cre}$*Apoc2*$^{fl/fl}$	Tamoxifen	1st round- 11 days- sacrificed
		Male (M)	1*Gt(ROSA)26Sor*$^{Cre/Cre}$*Apoc2*$^{fl/fl}$		
	WT	Male	1 CD45.2^{+}	Tamoxifen	
Experiment 2	*Apoc2*-KO	Male	1*Gt(ROSA)26Sor*$^{Cre/Cre}$*Apoc2*$^{fl/fl}$	Tamoxifen	1st round- 3 weeks- 2nd round- 1 week - sacrificed
	WT (6-month old)		1*Gt(ROSA)26Sor*$^{wt/wt}$*Apoc2*$^{fl/fl}$	Tamoxifen	
Experiment 3	*Apoc2*-KO	Male	2*Gt(ROSA)26Sor*$^{Cre/Cre}$*Apoc2*$^{fl/fl}$	Tamoxifen	1st round- 1 month- sacrificed
	WT		2 CD45.2^{+}	Tamoxifen	
Experiment 4	*Apoc2*-KO	Female	1*Gt(ROSA)26Sor*$^{Cre/Cre}$*Apoc2*$^{fl/fl}$	Tamoxifen	1st round- 1 month- sacrificed
		Male	1*Gt(ROSA)26Sor*$^{Cre/Cre}$*Apoc2*$^{fl/fl}$		
	WT	Female	1*Gt(ROSA)26Sor*$^{wt/wt}$*Apoc2*$^{fl/fl}$	Tamoxifen	
		Male	1*Gt(ROSA)26Sor*$^{wt/wt}$*Apoc2*$^{fl/fl}$		
Experiment 5	*Apoc2*-KO	Female	2*Gt(ROSA)26Sor*$^{Cre/Cre}$*Apoc2*$^{fl/fl}$	Tamoxifen	1st round- 6 weeks- sacrificed
	WT		2*Gt(ROSA)26Sor*$^{wt/wt}$*Apoc2*$^{fl/fl}$	Tamoxifen	
Experiment 6	*Apoc2*-KO	Male	3*Gt(ROSA)26Sor*$^{Cre/Cre}$*Apoc2*$^{fl/fl}$	Tamoxifen	1st round- 3 months- sacrificed
	WT		2*Gt(ROSA)26Sor*$^{wt/wt}$*Apoc2*$^{fl/fl}$	Tamoxifen	

Experiment 7	*Apoc2*-KO	Female	2*Gt(ROSA)26Sor*$^{Cre/Cre}$*Apoc2*$^{fl/fl}$	Tamoxifen	1st round- 5 months-sacrificed
	WT		2*Gt(ROSA)26Sor*$^{wt/wt}$*Apoc2*$^{fl/fl}$	Tamoxifen	1st round- 4 months-sacrificed
Experiment 8	*Apoc2*-KO	Female	2*Gt(ROSA)26Sor*$^{Cre/Cre}$*Apoc2*$^{fl/fl}$	Tamoxifen	1st round-3 weeks-weighted
		Male	2*Gt(ROSA)26Sor*$^{Cre/Cre}$*Apoc2*$^{fl/fl}$		
	WT	Female	2*Gt(ROSA)26Sor*$^{Cre/Cre}$*Apoc2*$^{fl/fl}$	Corn oil	
		Male	2*Gt(ROSA)26Sor*$^{Cre/Cre}$*Apoc2*$^{fl/fl}$		
Experiment 9	*Apoc2*-KO	Male	2*Gt(ROSA)26Sor*$^{Cre/Cre}$*Apoc2*$^{fl/fl}$	Tamoxifen	1st round- 6 months-sacrificed
	WT		2 CD45.2^{+}	Tamoxifen	
Experiment 10	*Apoc2*-KO	Female	2*Gt(ROSA)26Sor*$^{Cre/Cre}$*Apoc2*$^{fl/fl}$	Tamoxifen	1 round- bleed on Days 1, 7, 14, 22
		Male	2*Gt(ROSA)26Sor*$^{Cre/Cre}$*Apoc2*$^{fl/fl}$		
	WT	Female	3*Gt(ROSA)26Sor*$^{Cre/Cre}$*Apoc2*$^{wt/wt}$	Tamoxifen	
		Male	2*Gt(ROSA)26Sor*$^{Cre/Cre}$*Apoc2*$^{wt/wt}$		
Experiment 11	*Apoc2*-KO	Female	2*Gt(ROSA)26Sor*$^{Cre/Cre}$*Apoc2*$^{fl/fl}$	Tamoxifen	1st round- 5 months-sacrificed
		Male	2*Gt(ROSA)26Sor*$^{Cre/Cre}$*Apoc2*$^{fl/fl}$		1st round- 7 months-sacrificed
	WT	Female	2 CD45.2^{+}	Tamoxifen	1st round- 7 months-sacrificed
		Male	2 CD45.2^{+}		1st round- 5 months-sacrificed
Experiment 12	*Apoc2*-KO	Female	1*Gt(ROSA)26Sor*$^{Cre/Cre}$*Apoc2*$^{fl/fl}$	Tamoxifen	1st round- 15 months-sacrificed
		Male	1*Gt(ROSA)26Sor*$^{Cre/Cre}$*Apoc2*$^{fl/fl}$		1st round- 10 months-5-FU injection-4 days-sacrificed
	WT	Female	1 CD45.2^{+}	Tamoxifen	1st round- 15 months-sacrificed
		Male	1 CD45.2^{+}		1st round- 13 months-5-

					FU injection-4 days-sacrificed
Experiment 13	*Apoc2*-KO	Male (2 mice are 6-months old)	4*Gt(ROSA)26Sor*$^{Cre/Cre}$*Apoc2*$^{fl/fl}$	Tamoxifen	1st round- 1.5 years- bleed
	WT	Male	4*Gt(ROSA)26Sor*$^{Cre/Cre}$*Apoc2*$^{wt/wt}$	Tamoxifen	
Experiment 14	*Apoc2*-KO	Female	1*Gt(ROSA)26Sor*$^{Cre/Cre}$*Apoc2*$^{fl/fl}$	Tamoxifen	1st round- 1 month-bleeding
		Male	3*Gt(ROSA)26Sor*$^{Cre/Cre}$*Apoc2*$^{fl/fl}$		
	WT	Female	*2Gt(ROSA)26Sor*$^{wt/wt}$*Apoc2*$^{fl/fl}$	Tamoxifen	
		Male	1*Gt(ROSA)26Sor*$^{wt/wt}$*Apoc2*$^{fl/fl}$		
		Male	1CD45.2^{+}		

Table S2. Hematology analysis of blood samples from WT and *Apoc2*-KO mice

	WT mice (Mean ± SD)	*Apoc2*-KO mice (Mean ± SD)	P value (T-test)
White Blood Cell (x 10^3/µL)	5.043±1.706	5.400±2.416	0.768
Red Blood Cell (x 10^6/µL)	8.565±1.325	8.311±1.187	0.723
Hemoglobin (gr/dL)	13.55±1.979	13.19±1.467	0.710
Hematocrit (%)	39.60±5.807	38.51±4.992	0.724
Mean Corpuscular Volume (fL)	46.32±1.146	46.41±1.059	0.876
Mean Corpuscular Hemoglobin (pg)	15.80±0.5621	15.91±0.6067	0.866
Mean Corpuscular Hemoglobin Concentration (g/dL)	34.15±1.250	34.29±0.8934	0.824
Neutrophil (%)	19.88±3.916	17.17±5.637	0.344

Lymphocytes (%)	65.58±11.63	71.01±8.297	0.348
Monocytes (%)	5.433±1.356	5.271±2.077	0.874
Eosinophils (%)	6.500±7.326	4.886±3.545	0.809
Basophils (%)	2.600±2.647	1.657±1.127	0.427
Platelet (x 1000/µL)	528.3±352.0	628.1±397.2	0.644
Neutrophil (x 10^3/µL)	0.99331±0.3611	0.9757±0.6143	0.952
Lymphocytes (x 10^3/µL)	3.405±1.382	3.723±1.394	0.689
Monocytes (x 10^3/µL)	0.2667±0.09933	0.3086±0.2242	0.681
Eosinophils (x 10^3/µL)	0.2717±0.2105	0.2943±0.3414	0.891
Basophils (x 10^3/µL)	0.1067±0.06593	0.09857±0.09209	0.861

Table S3. The presence of bands related to different *Cre* genotypes

	Cre-MT primers	*Cre*-WT primers	Genotype
the presence of bands	+	-	Homozygous *Cre* gene (*Gt(ROSA)26Sor*$^{Cre/Cre}$)
	+	+	Heterozygous *Cre* gene (*Gt(ROSA)26Sor*$^{Cre/wt}$)
	-	+	WT (*Gt(ROSA)26Sor*$^{wt/wt}$)

Table S4. The presence of bands related to different *Apoc2 lox* sites genotypes

	Apoc2-lox primers	*Apoc2*-WT primers	Genotype
the presence of bands	+	-	Homozygous *Apoc2 lox* gene (*Apoc2*$^{fl/fl}$)
	+	+	Heterozygous *Apoc2 lox* gene (*Apoc2*$^{fl/wt}$)
	-	+	WT (*Apoc2*$^{wt/wt}$)

100
