# Supplementary material for: Establishment and functional analysis of an inducible Apoc2-knockout mouse model to investigate the role of Apoc2 in normal hematopoiesis
Source: Lab Anim (NY). 2026 Jul 10;55(9):354–66. doi: 10.1038/s41684-026-01748-z (PMC13518242; doi:10.1038/s41684-026-01748-z)
Supplement: Supplementary file 2 [file 41684_2026_1748_MOESM2_ESM.pdf]

nature portfolio

Corresponding author(s): Houda Alachkar

Last updated by author(s): Jul 11, 2025

Reporting Summary

Nature Portfolio wishes to improve the reproducibility of the work that we publish. This form provides structure for consistency and transparency in reporting. For further information on Nature Portfolio policies, see our Editorial Policies and the Editorial Policy Checklist.

Statistics

For all statistical analyses, confirm that the following items are present in the figure legend, table legend, main text, or Methods section.

n/a	Confirmed	
☐	☒	The exact sample size (*n*) for each experimental group/condition, given as a discrete number and unit of measurement
☐	☒	A statement on whether measurements were taken from distinct samples or whether the same sample was measured repeatedly
☐	☒	The statistical test(s) used AND whether they are one- or two-sided<br>*Only common tests should be described solely by name; describe more complex techniques in the Methods section.*
☐	☒	A description of all covariates tested
☐	☒	A description of any assumptions or corrections, such as tests of normality and adjustment for multiple comparisons
☐	☒	A full description of the statistical parameters including central tendency (e.g. means) or other basic estimates (e.g. regression coefficient) AND variation (e.g. standard deviation) or associated estimates of uncertainty (e.g. confidence intervals)
☒	☐	For null hypothesis testing, the test statistic (e.g. *F*, *t*, *r*) with confidence intervals, effect sizes, degrees of freedom and *P* value noted<br>*Give P values as exact values whenever suitable.*
☒	☐	For Bayesian analysis, information on the choice of priors and Markov chain Monte Carlo settings
☒	☐	For hierarchical and complex designs, identification of the appropriate level for tests and full reporting of outcomes
☒	☐	Estimates of effect sizes (e.g. Cohen's *d*, Pearson's *r*), indicating how they were calculated

Our web collection on statistics for biologists contains articles on many of the points above.

Software and code

Policy information about availability of computer code

Data collection	QuantStudio 12K Flex Software, BioTek Gen5 software, BD FACSDiva™ software, HeskaView™ Connect software
Data analysis	Graphpad, Micfrosoft Excel

For manuscripts utilizing custom algorithms or software that are central to the research but not yet described in published literature, software must be made available to editors and reviewers. We strongly encourage code deposition in a community repository (e.g. GitHub). See the Nature Portfolio guidelines for submitting code & software for further information.

Data

Policy information about availability of data

All manuscripts must include a data availability statement. This statement should provide the following information, where applicable:

- Accession codes, unique identifiers, or web links for publicly available datasets
- A description of any restrictions on data availability
- For clinical datasets or third party data, please ensure that the statement adheres to our policy

NA

March 2021

Field-specific reporting

Please select the one below that is the best fit for your research. If you are not sure, read the appropriate sections before making your selection.

☒ Life sciences ☐ Behavioural & social sciences ☐ Ecological, evolutionary & environmental sciences

For a reference copy of the document with all sections, see nature.com/documents/nr-reporting-summary-flat.pdf

Life sciences study design

All studies must disclose on these points even when the disclosure is negative.

Sample size	Sample size was determined based on literature review of similar studies in the filed , each experiment was repeated 2-3 times with different mice
Data exclusions	All data from technically proper experiment that lacked technincal errors were reported were reported
Replication	replications were described in the methods and the figure legends . each experiment were independently repeated 2-3 times at minimum.
Randomization	NA
Blinding	NA, no treatment or survival studies were involved.

Reporting for specific materials, systems and methods

We require information from authors about some types of materials, experimental systems and methods used in many studies. Here, indicate whether each material, system or method listed is relevant to your study. If you are not sure if a list item applies to your research, read the appropriate section before selecting a response.

Materials & experimental systems

n/a	Involved in the study	
☐	☒	Antibodies
☒	☐	Eukaryotic cell lines
☒	☐	Palaeontology and archaeology
☐	☒	Animals and other organisms
☒	☐	Human research participants
☒	☐	Clinical data
☒	☐	Dual use research of concern

Methods

n/a	Involved in the study	
☒	☐	ChIP-seq
☐	☒	Flow cytometry
☒	☐	MRI-based neuroimaging

Antibodies

Antibodies used	CD45.1 monoclonal antibody (A20) (Invitrogen Cat# 17-0453-81), CD45.2 monoclonal antibody (104) (Invitrogen Cat# 11-0454-81)
Validation	These are well validated antibodies by the manufacture and we use positive and negative control for each experiment.

Animals and other organisms

Policy information about studies involving animals; ARRIVE guidelines recommended for reporting animal research

Laboratory animals	C57BL/6 J mice (RRID:IMSR_JAX:000664),B6.SJL-Ptprca Pepcb/BoyJ mice (RRID:IMSR_JAX:002014) were ordered from Jackson laboratory. B6.129-Gt(ROSA)26Sortm1(cre/ERT2)Tyj/J (R26-CreERT2) mouse strain (RRID:IMSR_JAX:008463) homozygous for Cre gene (Gt(ROSA)26SorCre/Cre) were ordered from Jackson laboratory. C57/BL6 homozygous for the floxed Apoc2 gene mice (Apoc2fl/fl) were obtained from Dr. Alan Remaley's lab at the National Heart, Lung, and Blood Institute at the National Institute of Health. mice ages were ranged from 5 weeks to more than one year.
Wild animals	NA
Field-collected samples	NA
Ethics oversight	All animal studies were approved by the Institution for Animal Care and Use Committee (IACUC) at the University of Southern California (USC). Animal Protocol Number 20581.

Note that full information on the approval of the study protocol must also be provided in the manuscript.

Flow Cytometry

Plots

Confirm that:

☒ The axis labels state the marker and fluorochrome used (e.g. CD4-FITC).

☒ The axis scales are clearly visible. Include numbers along axes only for bottom left plot of group (a 'group' is an analysis of identical markers).

☒ All plots are contour plots with outliers or pseudocolor plots.

☒ A numerical value for number of cells or percentage (with statistics) is provided.

Methodology

Sample preparation	described in the methods:For the CellTrace assay, about 100 L cells were collected from every well to run the flow cytometry. The reduced fluorescent intensities represented the CellTrace™ CFSE dye dilution tracked by flow cytometry. Flow cytometry analysis was also used to assess the BM engraftment in competitive repopulation assay. BM, spleen and blood cells were collected from CD45.1+CD45.2+ heterozygous recipient mice. 100 L PBS was used to prepare the dye solution with 1.25 L CD45.1 monoclonal antibody (A20) (Invitrogen Cat# 17-0453-81), and 0.5 L CD45.2 monoclonal antibody (104) (Invitrogen Cat# 11-0454-81) for each sample. Cells were incubated on ice protected from light for 30 minutes. Cells were then washed with PBS three times and then measured the conjugation of APC for CD45.1 and FITC for CD45.2 by flow cytometry.
Instrument	Flow Cytometer – BD Fortessa X20
Software	FlowJo
Cell population abundance	NA, no sorting was done
Gating strategy	We provide these in supplemental data along with the The axis labels state the marker and fluorochrome used

☒ Tick this box to confirm that a figure exemplifying the gating strategy is provided in the Supplementary Information.
